# Cooperatively rearranging regions change shape near the mode-coupling crossover for colloidal liquids on a sphere

**DOI:** 10.1038/s41467-020-18760-7

**Published:** 2020-10-02

**Authors:** Navneet Singh, A. K. Sood, Rajesh Ganapathy

**Affiliations:** 1 Chemistry and Physics of Materials Unit, Jawaharlal Nehru Centre for Advanced Scientific Research, Jakkur, Bangalore, 560064 India; 2 Department of Physics, Indian Institute of Science, Bangalore, 560012 India; 3 International Centre for Materials Science, Jawaharlal Nehru Centre for Advanced Scientific Research, Jakkur, Bangalore, 560064 India; 4 School of Advanced Materials (SAMat), Jawaharlal Nehru Centre for Advanced Scientific Research, Jakkur, Bangalore, 560064 India

**Keywords:** Colloids, Glasses, Condensed-matter physics, Structure of solids and liquids, Statistical physics

## Abstract

The structure and dynamics of liquids on curved surfaces are often studied through the lens of frustration-based approaches to the glass transition. Competing glass transition theories, however, remain largely untested on such surfaces and moreover, studies hitherto have been entirely theoretical/numerical. Here we carry out single particle-resolved imaging of dynamics of bi-disperse colloidal liquids confined to the surface of a sphere. We find that mode-coupling theory well captures the slowing down of dynamics in the moderate to deeply supercooled regime. Strikingly, the morphology of cooperatively rearranging regions changed from string-like to compact near the mode-coupling crossover—a prediction unique to the random first-order theory of glasses. Further, we find that in the limit of strong curvature, Mermin–Wagner long-wavelength fluctuations are irrelevant and liquids on a sphere behave like three-dimensional liquids. A comparative evaluation of competing mechanisms is thus an essential step towards uncovering the true nature of the glass transition.

## Abstract (web summary)

The static and dynamic behavior of condensed phases residing on curved surfaces can be fundamentally different from their counterparts in Euclidean space. Singh et al. test several competing glass theories on colloidal liquids confined to the surface of a sphere and show they behave like 3D bulk liquids.

## Introduction

Condensed phases can acquire a new life when confined to curved surfaces^1–4^. For instance, the low temperature phase of a single-component system on the hyperbolic plane is a glass and not a crystal, like in flat space, since curvature introduces an irreducible number of topological defects^5,6^. Alternatively, while glassy dynamics in many metallic liquids is thought to stem from the inability of the locally favored structure—an icosahedron—to proliferate in flat space, these fivefold symmetric motifs under certain conditions can perfectly tile the 3-sphere *S*^3^, ref. ^7^. Curving space can thus promote or alleviate frustration^8^. However, how liquids vitrify on curved surfaces have hitherto been investigated only in theory and numerics and has never been interrogated through experiments. Further, and not surprisingly, almost all these theoretical/numerical studies are in the context of frustration-based approaches to the glass transition and to what extent competing theories capture glass transition phenomenology on such surfaces remains largely unexplored. It is only recently that numerical studies of a single-component liquid on a sphere, the simplest curved surface and denoted as *S*^2^, found that mode-coupling theory (MCT) can qualitatively capture the role of curvature on glassy dynamics^9^. The power-law scaling of the structural relaxation time, *τ*_*α*_, near the mode-coupling singularity and the nature of the singularity were also found to be identical to that in Euclidean space. Subsequent experiments, however, revealed that at high densities, liquids of monodisperse particles on *S*^2^ do not vitrify but instead freeze into a defect-ordered single crystal^4^. Freezing can be avoided by working with liquids of bidisperse particles. MCT is yet to be tested on such liquids on *S*^2^, and doing so opens the possibility of examining if the relaxation processes envisaged by the thermodynamic framework of the random first-order transition theory (RFOT) of glasses are at play^10^. Since MCT is a mean-field theory, the singularity predicted by this theory is only a crossover in finite dimensions and across this crossover RFOT anticipates a change in the shape of cooperatively rearranging regions (CRRs) from string-like to compact^11^. This prediction, unique to RFOT, has been validated in simulations and experiments in Euclidean space^12–16^. Whether curving space fundamentally alters this scenario is not known.

Probing the dynamics of liquids on curved surfaces can also help address another key issue. There is now a consensus that in *d* ≤ 2 dimensions, supercooled liquids, and glasses, like crystals, are affected by long-wavelength Mermin–Wagner (MW) fluctuations^17–19^. These fluctuations provide an additional channel for structural relaxation and transient particle localization, a signature feature of glassy dynamics in 3-dimensions, is absent. In fact, even in the normal liquid regime where such localization effects are absent, while in *d* = 3 the long-time diffusion constant $D\propto {\tau }_{\alpha }^{-\kappa }$ with *κ* = 1, in *d* = 2 MW fluctuations result in an anomalous scaling with *κ* > 1^20^. MW fluctuations are also strongly system size-dependent and their effects diminish systematically on reducing system size^17–21^. In fact, for liquids on closed surfaces, like *S*^2^, system size and curvature effects are intertwined^22^. The role of MW fluctuations on the dynamics of liquids on curved surfaces, let alone on *S*^2^, is not known.

Motivated by these open issues and the lack of experiments that have attempted to address them, here we investigated dynamical slowing down of colloidal liquids, at the single-particle level, on a sphere. We not only found that standard liquid state theory quantitatively describes the structure of liquids on *S*^2^ but also shows that MCT successfully captures the growth in *τ*_*α*_ in the vicinity of the mode-coupling glass transition. Strikingly, on approaching the MCT singularity from the liquid side, we find that the shapes of CRRs evolve from being string-like to compact as anticipated within the RFOT. By probing dynamics using both conventional and cage-relative relaxation measures, we show that in the limit of strong curvature, MW fluctuations do not significantly influence dynamics and liquids on *S*^2^ behave like liquids in *d* = 3.

## Results

### Structure of supercooled liquids and glasses on the surface of a sphere

Our experimental system comprised of bidisperse charged hydrophobic colloids that were bound to the interface between spherical oil droplets and an aqueous phase due to image charge forces^2^ (Supplementary Fig. 1 and Supplementary Movie 1). The particle charge and its image constitute an electric dipole and the colloids interact through repulsive dipolar forces (see “Methods” section for details). Due to poor screening of the particle charge in the oil phase, which is the suspending fluid for the particles, interactions are long ranged and soft. By starting with different initial particle number densities in the oil phase, we systematically tuned the areal density of particles, *n*, adsorbed at the interface. Single-particle dynamics were imaged using a confocal microscope at maximum possible temporal resolution (Leica TCS SP8 II, 63× oil-immersion objective) (see “Methods” section for details and Supplementary Note 1). Previous studies have found that the complete phase behavior of this system can be parametrized through a single dimensionless parameter, Γ, which is the ratio of the total electrostatic dipole and thermal energies^4,18^. For a bidisperse system, Γ $=\frac{{(\pi n)}^{3/2}}{8\pi \epsilon {k}_{B}T}{({\xi }_{b}{p}_{b}+(1-{\xi }_{b}){p}_{s})}^{2}$, where *ϵ* is the dielectric constant of the suspending fluid, here the oil phase, *ξ*_*b*_ is the fraction of big particles and *p*_*b*_ and *p*_*s*_ are the dipole moments of the big and small particles, respectively. We directly determined Γ using the radial pair-correlation function method^23^. Although the oil phase does not exactly match the particle density, gravitational effects are negligible. In all our experiments the size of the oil droplet was nearly a constant and had a typical radius of curvature *R* = (19.2 ± 1.2)*r*, where $r=\frac{{r}_{b}+{r}_{s}}{2}$. Here, *r*_*b*_ = 0.908 μm and *r*_*s*_ = 0.755 μm are the hard-sphere radii of the big and small colloids, respectively.

**Fig. 1:**
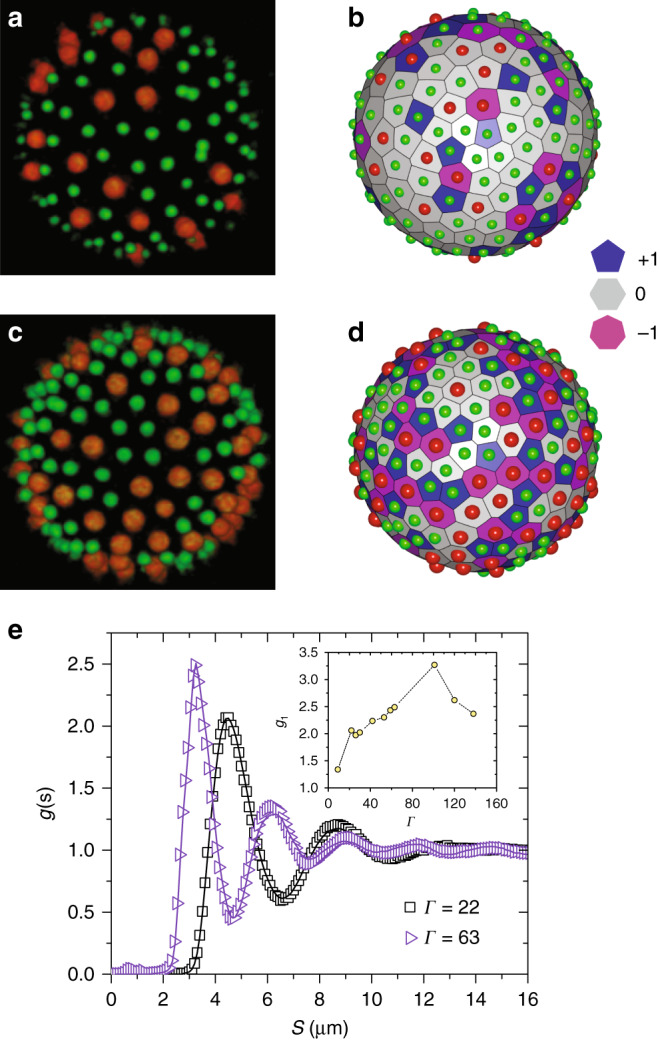
Static structure of supercooled liquids on a sphere. **a**, **c** 3D Confocal micrographs of bidisperse colloidal liquids wrapped on the surface of *S*^2^ at two areal densities. The red and the green colors represent big and small particles which are of radius *σ*_*b*_ = 0.908 μm and *σ*_*s*_ = 0.755 μm, respectively. **b**, **d** Radical Voronoi tessellations of particle packings shown in (**a**) and (**c**). Blue, gray, and magenta polygons are associated with 5, 6, and 7 coordinated particles, respectively. These Voronoi tessellations show a disordered phase with topological defects uniformly covering the surface. **e** Pair-correlation function *g*(*s*) as a function of the geodesic distance *s* for Γ = 22 (black open squares) and Γ = 63 (violet open triangles), displaying liquid-like short-range order. The lines are hypernetted— chain (HNC) fit to the data, indicating good agreement between theory and experiment. Inset to (**e**) shows nonmonotonic evolution of the height of the first peak (*g*_1_) of *g*(*s*) as a function of Γ.

  Figure 1a, c shows representative snapshots of the reconstructed upper hemisphere of particle-laden droplets at low and high Γ, respectively. For monodisperse particles with isotropic interactions, the preferred coordination in 2D-flat space is six^24^. Frustration introduced through spatial curvature and particle size bidispersity result in coordination defects, i.e., particles having a local coordination number different from six, which act as topological charges. Although topology sets the net charge, 12 for *S*^2^, it neither constrains the total number of these charges nor does it determine their arrangement. To gain insights into how these defects are arranged in our system, we tessellated space using the radical Voronoi method that is appropriate for bidisperse packings^25,26^. The tessellations corresponding to Fig. 1a and c are shown in Fig. 1b and d, respectively. The Voronoi cells in Fig. 1b and d are colored according to their topological charge (6 minus the local coordination of the particle in the cell). Hexagonal cells (shown in gray) have zero charge, while pentagonal (shown in blue) and heptagonal cells (shown in purple), which are the most frequently occurring defects, have a charge of  +1 and  −1, respectively. Fourfold and eightfold coordinated defects do occasionally appear, but these are rare as they are energetically costly (Supplementary Fig. 2). At all Γ studied, neither did we observe any semblance of crystalline order nor did we observe any demixing with time (Supplementary Fig. 3) and the topological defects uniformly covered the surface. The net topological charge of 12, however, was entirely from pentagonal defects. For large Γ (Fig. 1d), we observed defects organized into chains that were branched and occasionally also formed closed loops as seen in a recent numerical study of binary mixtures on *S*^2^, ref. ^27^ (Supplementary Fig. 2). This is unlike what is observed for monodisperse particles at large Γ, where the 12 excess pentagons are localized at the vertices of an icosahedron and the defect chains—also called scars—are linear and along its edges^4^. Despite the very soft nature of the pair-potential, particle bidispersity clearly thwarts the occurrence of the crystalline ground state.

The short-range of structural correlations, as is expected for a liquid, is evident from the radial pair-distribution function *g*(*s*) which is plotted as a function of the geodesic distance *s* in Fig. 1e (Supplementary Note 2). In Euclidean space, the hypernetted chain (HNC) approximation has been found to well-capture the structure of liquids when particle interactions are soft^28^. We found this to be true even for liquids on *S*^2^ (lines in Fig. 1e are HNC fits to *g*(*s*)) allowing us to directly calculate the particle pair-potential and hence Γ (Supplementary Note 3). An exponential fit to the envelope of *g*(*s*) yielded the correlation length which even at large Γ is only  ≈2*σ*, where *σ* is the position of first peak of *g*(*s*) (Supplementary Fig. 5). Simulations of monodisperse particles with Lennard–Jones interactions on *S*^2^ found a much larger correlation length of almost 5*σ* possibly due to the presence of a hexatically ordered phase at low temperatures. Since particle interactions are soft, the position of the first peak in *g*(*s*) moved to smaller *s* with increasing Γ. Interestingly, the height of the first peak of *g*(*s*), *g*_1_, showed a nonmonotonic evolution with Γ (inset to Fig. 1e). A maximum in *g*_1_ has been observed at the jamming density in fluids of soft particles in Euclidean space and arises due to competing contributions from entropy and energy^29–31^. On approaching the jamming density from below, the system behaves like a hard-sphere fluid and *g*_1_ grows systematically with density. Since particle overlaps for soft spheres are not energetically costly, for densities beyond the jamming density the system can gain free energy by maximizing disorder and permitting overlaps and *g*_1_ begins to decrease with a maximum at jamming. Dense liquids of soft particles on *S*^2^ thus show the same structural anomaly as their counterparts do in Euclidean space.

### Dynamics of supercooled liquids and glasses on a sphere

**Fig. 2:**
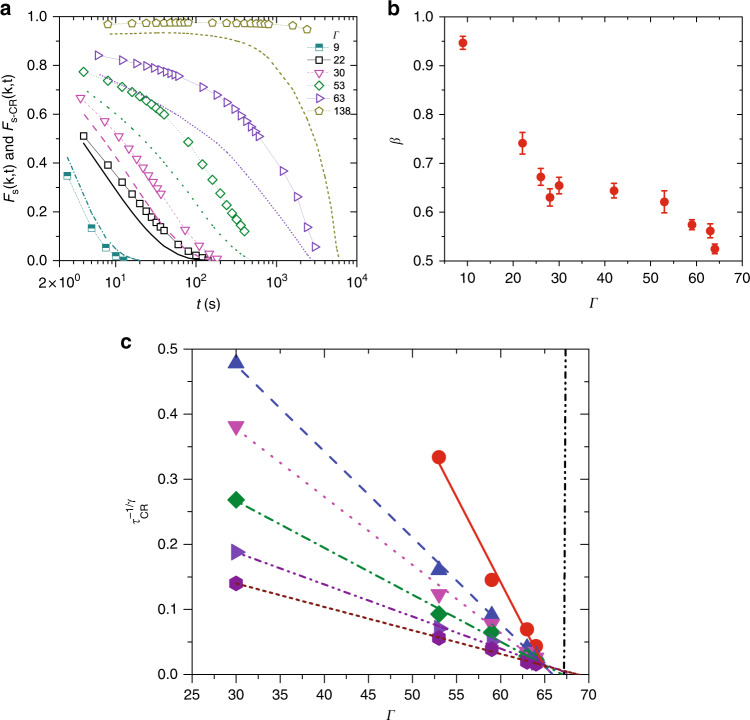
Testing predictions of MCT on a sphere. **a** The normal self-intermediate scattering functions *F*_*s*_(*k*, *t*) (lines) and the cage-relative self-intermediate scattering function *F*_*s*−CR_(*k*, *t*) (symbols) for various Γ values. At low Γ, since the cage around the particle is itself quite mobile, when computing the cage-relative measure, a random displacement due to cage motion also contributes to the particle displacement over and above that due to its Brownian motion. Thus, *F*_*s*−CR_(*k*, *t*) decays faster than *F*_*s*_(*k*, *t*). As Γ is increased and the cages become more rigid, this effect diminishes and the decay of *F*_*s*−CR_(*k*, *t*) is slower than *F*_*s*_(*k*, *t*). **b** Variation of the stretching exponent *β*, obtained from fits to *F*_*s*−CR_(*k*, *t*), with Γ. The error bars represent the standard error (SE) and are obtained from fits to the data. **c** ${\tau }_{\mathrm{CR}}^{-1/\gamma }$ versus Γ for various wavevectors *k*. *k* = 3.69 (red circles), *k* = 2.99 (blue triangles), *k* = 2.73 (magenta triangles), *k* = 2.32 (olive green squares), *k* = 1.90 (violet triangles), and *k* = 1.53 (wine hexagons). The vertical black line shows the mode-coupling glass transition. The power-law exponent was found to be *γ* = 1.89.

We now turn our attention to dynamics. We first computed the self-intermediate scattering function which on a sphere is defined as ${F}_{s}(k,t)=\frac{1}{M}\mathop{\sum }\nolimits_{j = 1}^{M}\left\langle {P}_{kR}\left(\cos \left(\frac{\Delta {s}_{j}(0,t)}{R}\right)\right)\right\rangle$^9,22^. Here, *P*_*kR*_ is the Legendre polynomial with *kR* being rounded-off to the nearest-integer, *k* is the wavevector corresponding to the first peak of *g*(*s*), *M* is the number of particles and Δ*s*_*j*_(0, *t*) is the geodesic displacement of particle *j* over time *t*. At large supercooling and due to the very soft nature of the pair-potential, particles can undergo substantial collective displacements without any cage-breaking and this can lead to the decay of *F*_*s*_(*k*, *t*). These effects may be further exacerbated by MW fluctuations, which have been found to be more pronounced for soft than for hard potentials^32^. Since the *α*—relaxation process necessarily involves particles breaking out of their confining cages, we computed the cage-relative self-intermediate scattering function *F*_*s*−CR_(*k*, *t*) which is sensitive only to this process^17–21^. Calculating *F*_*s*−CR_(*k*, *t*) involves replacing Δ*s*_*j*_(0, *t*) in the definition of *F*_*s*_(*k*, *t*) with the cage-relative displacement, Δ*s*_*j*−CR_(0, *t*), where $\Delta {s}_{j-\mathrm{CR}}(t)=\Delta {s}_{j}(0,t)-\frac{1}{\mathrm{N{N}}_{j}}\mathop{\sum }\nolimits_{i = 1}^{\mathrm{N{N}}_{j}}({s}_{i}(t)-{s}_{i}(0))$. Here, NN_*j*_ is the number of nearest-neighbors of central particle *j* and the second term is just the average displacement of the cage over time *t* (Supplementary Note 4). Figure 2a shows *F*_*s*_(*k*, *t*) (dashed lines) and *F*_*s*−CR_(*k*, *t*) (symbols) versus *t* for various Γ. We did not observe any aging behavior over the duration of our experiments (Supplementary Fig. 7) and this allowed us to unambiguously define *τ*_*α*_ and ${\tau }_{\alpha }^{\mathrm{CR}}$ as ${F}_{s}(k,{\tau }_{\alpha })=\frac{1}{e}$ and ${F}_{s-\mathrm{CR}}(k,{\tau }_{\alpha }^{\mathrm{CR}})=\frac{1}{e}$, respectively. The rapid slowing down of dynamics with Γ is apparent. More importantly, while at small Γ the decay profiles *F*_*s*_(*k*, *t*) and *F*_*s*−CR_(*k*, *t*) are more or less similar, the discrepancy between them grows with increasing Γ and for Γ ≥ 63, *F*_*s*−CR_(*k*, *t*) does not decay while *F*_*s*_(*k*, *t*) does. This suggests that a substantial contribution to the decay of *F*_*s*_(*k*, *t*) stems from processes that does not lead to changes to the local connectivity of the particles. Focusing on *F*_*s*−CR_(*k*, *t*), we observed that the *α*—relaxation process becomes an increasingly stretched exponential relaxation with undercooling, as is typical of liquids approaching the glass transition in Euclidean space, and the stretching exponent *β*_CR_ decreases at small Γ and develops a plateau at intermediate Γ before decreasing again near Γ_MCT_ (Fig. 2b)^33,34^.

We next determined if the slowing down of dynamics with supercooling can be captured by MCT^35^. When either the particle volume/area fraction, *ϕ*, or the temperature, *T*, is the parameter that controls dynamical slowing down, as is usually the case, MCT anticipates ${\tau }_{\alpha }\propto {(C-{C}_{\mathrm{MCT}})}^{-\gamma }$. Here, *C* plays the role of either *ϕ* or *T*, *C*_MCT_ is the location of mode-coupling glass transition and *γ* is a scaling exponent which ranges from 1.5 to 3 depending on the system under consideration^33,34^. For our dipolar colloidal liquids, we carried out the MCT scaling analysis with Γ as the control parameter (Fig. 2c). Most remarkably, the growth of *τ*_*α*_ with supercooling indeed follows a power-law (*γ* =  1.8), a value consistent with MCT predictions, and the curves at various *k*s all yield a nearly identical mode-coupling glass transition with Γ_MCT_ = 67 ± 1. The quality of the scaling was very poor when *ϕ* was chosen to be the control parameter reaffirming that it is Γ that controls dynamics in our system.

### Change in morphology of CRR’s near MCT crossover

**Fig. 3:**
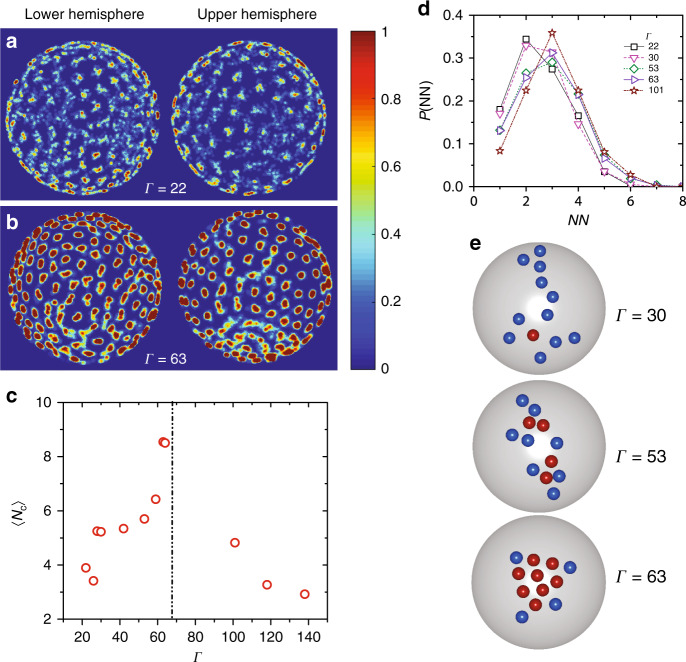
Change in shape of CRRs on approaching the MCT crossover. **a**, **b** Particle displacement map over the cage-breaking time t* for Γ = 22 and Γ = 63, respectively. Here colors represent overlap values with red representing high particle overlap over t* and blue representing poor overlaps indicating particles having made hop. **c** Average cluster size 〈*N*_*c*_〉 verses Γ (red open circles), 〈*N*_*c*_〉 shows a maximum at mode-coupling crossover Γ_MCT_(vertical black dash-dot line). **d** Distribution of the number of fast nearest-neighbors for a mobile particle, *P*(NN), for Γ = 22 (black squares), Γ = 30 (magenta triangles), Γ = 53 (olive green squares), Γ = 63 (violet triangles), and Γ = 101 (wine stars). The peak of *P*(NN) shifts from NN = 2 to NN = 3 indicating that dynamical heterogeneities becoming more compact with increasing Γ. **e** Representative dynamical heterogeneities of 12-particle clusters for Γ = 30, 53, and 63. Particles that comprise the core of dynamical heterogeneities are shown in red while the stringy particles are shown in blue.

An increasingly stretched exponential relaxation with supercooling is often attributed to increasingly heterogeneous dynamics. To quantify these dynamical heterogeneities, we first calculated the cage-relative non-Gaussian parameter, ${\alpha }_{2}^{\mathrm{CR}}(t)$ using a definition appropriate for *S*^2^. Particle dynamics are maximally non-Gaussian over the cage-breaking time t* and this is manifest as a peak in ${\alpha }_{2}^{\mathrm{CR}}(t={t}^{* })$ (Supplementary Note 5 and Supplementary Fig. 9). Figure 3a and b show the particle displacement maps over t* for a low and high Γ value, respectively. The colors represent the extent to which a particles’ position overlapped with itself over t* with red representing high particle overlap (immobile particle) and blue representing poor overlap (mobile particle). While at low Γ, the dynamics appears homogeneous as is typical of the high temperature/low density liquid state, at the larger Γ, much of the system appears frozen with only a few particles undergoing large displacements (Supplementary Movies 3, 4 & Supplementary Fig. 10). Moreover, the dynamics was also highly cooperative with the mobile particles being spatially clustered^36^. Further, unlike in simulations of liquids of monodisperse particles on *S*^2^, where dynamical heterogeneities were found to be largely localized at topological defects at low temperatures^22^, the heterogeneities in our system appear at spatially random locations and are reminiscent of standard supercooled liquid dynamics in Euclidean space^33^. Next, we identified the top 10% of the most-mobile particles over t* and clustered them if their centers were within 1.4*σ* of each other. From the probability distribution of cluster sizes *P*(*n*), we calculated the average cluster size of CRRs $\langle {N}_{c}\rangle =\frac{{\sum }_{n}{n}^{2}P(n)}{{\sum }_{n}nP(n)}$ at all Γs studied. 〈*N*_*c*_〉 increases with Γ until Γ_*g*_ beyond which it sharply decreases (Fig. 3c). 〈*N*_*c*_〉 has been found to decrease beyond *ϕ*_*g*_ in hard particle liquids and has been attributed due to cooperative dynamics associated with the short-time *β*-relaxation process^14,16,36^. A similar mechanism may also be at play here. However, unlike in hard-sphere liquids where *τ*_*α*_ diverges at *ϕ*_*g*_, in soft particle liquids it stays finite^31^. Whether the maximum in 〈*N*_*c*_〉 at Γ_*g*_ in our system shares the same mechanistic origin as in hard particle liquids remains to be seen.

According to RFOT, the mode-coupling singularity is only a dynamical crossover in finite dimensions^10^. RFOT further posits CRRs to be composite objects that comprise of a compact core surrounded by a ramified string-like shell^11^. While at mild supercooling CRRs are predominantly string-like and grow in length with increasing supercooling, near and beyond the mode-coupling glass transition, the string-length decreases and the compact core begins to dominate their overall morphology. A change in the shape of dynamical heterogeneities on approaching the MCT singularity has indeed been observed in both numerical and experimental studies on liquids with^12,14^ and without^13,15,16^ quenched disorder. To determine if this was the case for liquids on *S*^2^ as well, we computed the distribution of mobile nearest-neighbors *P*(NN) to a mobile particle^14^. *A P*(NN) that is peaked at NN = 2 indicates more string-like CRRs with mobile particles predominantly have one neighbor ahead and behind them, while a *P*(NN) that is peaked at NN > 2 indicates more compact CRRs. Most remarkably, with Γ the peak of *P*(NN) shifts from NN = 2 to NN = 3 (Fig. 3d). To illustrate that the shifting of the peak of *P*(NN) is not a trivial outcome of a growing 〈*N*_*c*_〉, with Γ, we analysed the morphology of CRRs of a fixed size of 12 particles (Fig. 3e). We identified the core and the shell particles of the CRRs, shown as red and blue spheres, respectively, following^14^. As anticipated by RFOT, CRRs indeed have a core-shell structure with the string-like shell dominating their morphology at low Γ and the compact core dominating it at high Γ.

### Mermin–Wagner fluctuations on the surface of a sphere

**Fig. 4:**
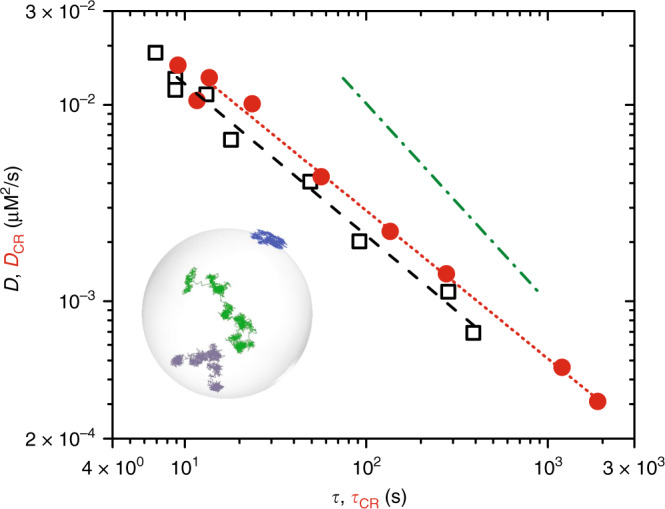
Liquids on *S*^2^ behave like 3D liquids in the limit of strong curvature. The figure shows the diffusion coefficient *D* versus *τ* (open black squares) and *D*_CR_ versus *τ*_CR_ (red filled circles) for supercooled liquids on the surface of a sphere scale. The exponent in both cases is nearly same with *κ* ≈ *κ*_CR_ ≈ 0.75. The green dash-dot line is a guide to the eye and indicates slope 1. The inset to the figure shows particle trajectories on the sphere displaying intermittent caging with abrupt hops. This behavior is typical of deeply supercooled liquids in 3D Euclidean space^17^.

The presence of dynamical heterogeneities in the supercooled regime implies that *D* is no longer proportional to *τ*_*α*_, like in simple liquids, but instead scales as $D\propto {\tau }_{\alpha }^{-\kappa }$ with *κ* < 1. Recent studies found that besides dynamical heterogeneities, MW fluctuations, which are prevalent in 2D, also contribute to altering the value of *κ*^20^. This implies that when MW fluctuations are removed using cage-relative measures, the exponent *κ*_CR_ connecting the cage-relative diffusivity *D*_CR_ and ${\tau }_{\alpha }^{\mathrm{CR}}$ is different from *κ* with 1 ≥ *κ*_CR_ > *κ* in the supercooled regime. In 3D liquids, where MW fluctuations have a lesser role, *κ*_CR_ ≈ *κ*^20^. A change in the value of exponent between the normal and the cage-relative measures thus serves as a readout for the presence of MW fluctuations. We calculated *D* and *D*_CR_ from the normal and the cage-relative mean-squared displacement at various Γs (Supplementary Fig. 8). Figure 4 shows a log–log plot of *D*_CR_ versus ${\tau }_{\alpha }^{\mathrm{CR}}$ (circles) and *D* versus *τ*_*α*_ (squares). The exponents *κ*_CR_ and *κ* are almost identical suggesting that in the limit of strong curvature ($\frac{R}{\sigma }\approx 4$), studied here, MW fluctuations are irrelevant and that these liquids behave like liquids in 3D. This observation is also supported by the inset to Fig. 4, where we find particle trajectories clearly showing transient particle localization without correcting for cage-displacements (Supplementary Movie 5). Such localization effects would have been absent had significant MW fluctuations been present^17^.

## Discussion

Collectively, our experiments show for the first time that there are striking parallels between the behavior of supercooled colloidal liquids on a sphere and those in Euclidean space. By using a bidisperse colloidal liquid to suppress the defect-ordered crystal phase at high densities^4^, we have shown that standard liquid state theory can quantitatively describe the structure of liquids on *S*^2^. We not only found that MCT can describe dynamical slowing down but also show that the shapes of CRRs changes from string-like to compact near the MCT singularity. The latter finding in particular is unique to RFOT^11^ and cannot be explained, as yet, by other theories including kinetic approaches such as the dynamical facilitation theory the glass transition^34^. Whether our findings carry over to surfaces with negative curvature remain to be seen. A natural step moving forward would be to examine predictions of inhomogeneous MCT^37,38^ and also track the evolution of static and dynamic correlation lengths with supercooling on curved surfaces. Introducing quenched disorder by pinning particles may prove useful, like in flat space, in helping prune glass transition theories^12,34^. Our observation that MW fluctuations do not contribute to the dynamics is possibly an immediate consequence of the finiteness of a closed surface. Since no boundary conditions need to be defined for a closed surface, our experiments pave the way for studying the role of finite-size effects on glassy dynamics^39^.

## Methods

### Experimental details

Our experimental system was a binary mixture of Poly methylmethacrylate (PMMA) colloids of radii *r*_*b*_ = 0.908 μm, *r*_*s*_ = 0.755 μm (size ratio *r*_*b*_/*r*_*s*_ = 1.20). Initially, the colloidal particles are suspended in a low dielectric constant oil mixture composed of cyclohexyl bromide (*ε*_CXB_ = 7.9) (65.5% v/v), and decalin (*ε*_Dec_ = 2.23) (34.5% v/v). The oil phase matches the refractive index and density of the PMMA colloidal particles. We added 100 μl of the PMMA-in-oil colloids to a 1 ml solution of glycerol and water (*ε*_Gly_ = 42.5, *ε*_Water_ = 80.4) (50% v/v) in a micro-centrifuge tube, which was manually shaken to create the oil droplets. The manual shaking results in emulsion droplets sizes ranging from 10 to 200 μm in radius. We focused our attention on emulsion droplets of radius *R* = (19.2 ± 1.2)*r*, where $r=\frac{{r}_{b}\,+\, {r}_{s}}{2}$. The PMMA particles in oil droplets become charged due to the dissociation of cyclohexyl bromide into H^+^ and Br^−^, refs. ^4,40^. These charged PMMA particles are drawn toward oil-aqueous interface due to the formation of image charges of opposite sign in the aqueous phase (Supplementary Fig. 1). A charge *q* embedded in a dielectric (*ε*_Oil_) at a distance *d* away from a planar interface of another dielectric (*ε*_Aqueous_) will form an image charge *q*_image_^41^:

1 $${q}_{{\rm{image}}}=-\frac{({\varepsilon }_{{\rm{Aqueous}}}-{\varepsilon }_{{\rm{Oil}}})}{({\varepsilon }_{{\rm{Aqueous}}}+{\varepsilon }_{{\rm{Oil}}})}q.$$

We filled the requisite amount of the oil-aqueous emulsion in an open cylindrical cell. Oil droplets (1.18 g cm^−3^) are denser than the aqueous phase (1.1 g cm^−3^) and sediment to the bottom of the cell. The dynamics of bi-dispersed PMMA particles at the oil-aqueous interface was observed using a confocal microscope (Leica SP8 with a 63× oil-immersion objective, N.A. 1.4). The images acquired from the confocal microscope were processed and rendered using ImageJ and Matlab, and the center-of-mass coordinates of the particles were obtained using standard Matlab algorithms (Supplementary Movie 2)^42^. Further analysis was done using custom codes written in Matlab.

## Supplementary information

Supplementary Information

Peer Review File

Description of Additional Supplementary Files

Supplementary Movie 1

Supplementary Movie 2

Supplementary Movie 3

Supplementary Movie 4

Supplementary Movie 5

## Data Availability

The source data sets generated for the current study are available from the corresponding author upon reasonable request.
