# Supplementary material for: Cooperatively rearranging regions change shape near the mode-coupling crossover for colloidal liquids on a sphere
Source: Nat Commun. 2020 Oct 2;11:4967. doi: 10.1038/s41467-020-18760-7 (PMC7532192; doi:10.1038/s41467-020-18760-7)
Supplement: Supplementary file 1 — Supplementary Information [file 41467_2020_18760_MOESM1_ESM.pdf]

Online Supplementary Material for

Cooperatively Rearranging Regions Change Shape Near the Mode-coupling Crossover for Colloidal Liquids on a Sphere

Navneet Singh*,[1] A K Sood,[2,3] and Rajesh Ganapathy[3,4]

[1] *Chemistry and Physics of Materials Unit, Jawaharlal Nehru Centre for Advanced Scientific Research, Jakkur, Bangalore - 560064, INDIA*

[2] *Department of Physics, Indian Institute of Science, Bangalore- 560012, INDIA*

[3] *International Centre for Materials Science, Jawaharlal Nehru Centre for Advanced Scientific Research, Jakkur, Bangalore - 560064, INDIA*

[4] *School of Advanced Materials (SAMat), Jawaharlal Nehru Centre for Advanced Scientific Research, Jakkur, Bangalore - 560064, INDIA*

* Corresponding author

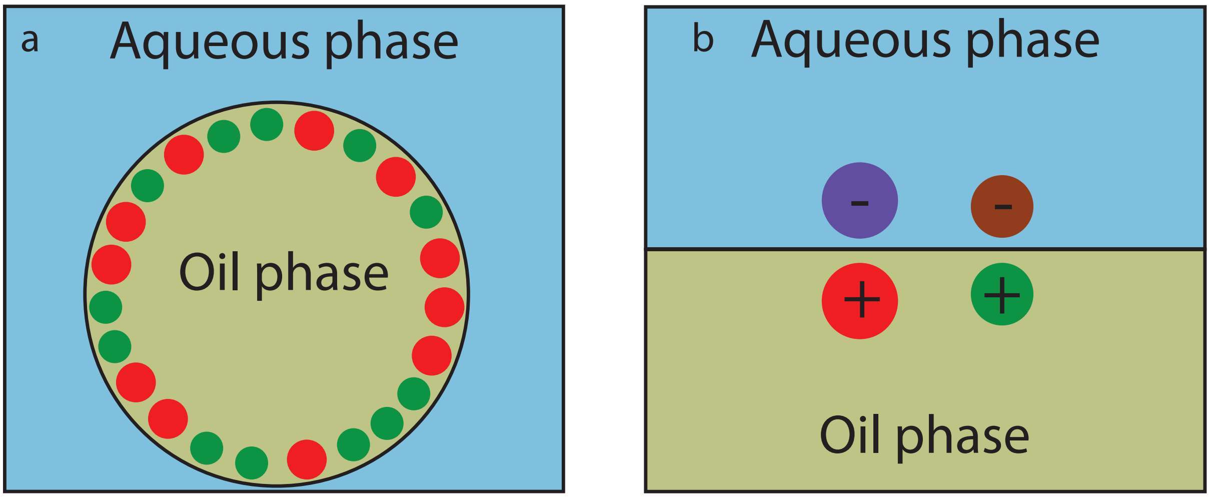

Supplementary Figure 1: **Experimental geometry and image charge formation.** **(a)** Pictorial representation of the experimental system of bi-disperse PMMA colloids on the interface of an oil-aqueous emulsion droplet. **(b)** Schematic representation of image charge formation at an oil-aqueous interface, a charged particle in the oil phase induces an image charge of opposite sign in the aqueous phase.

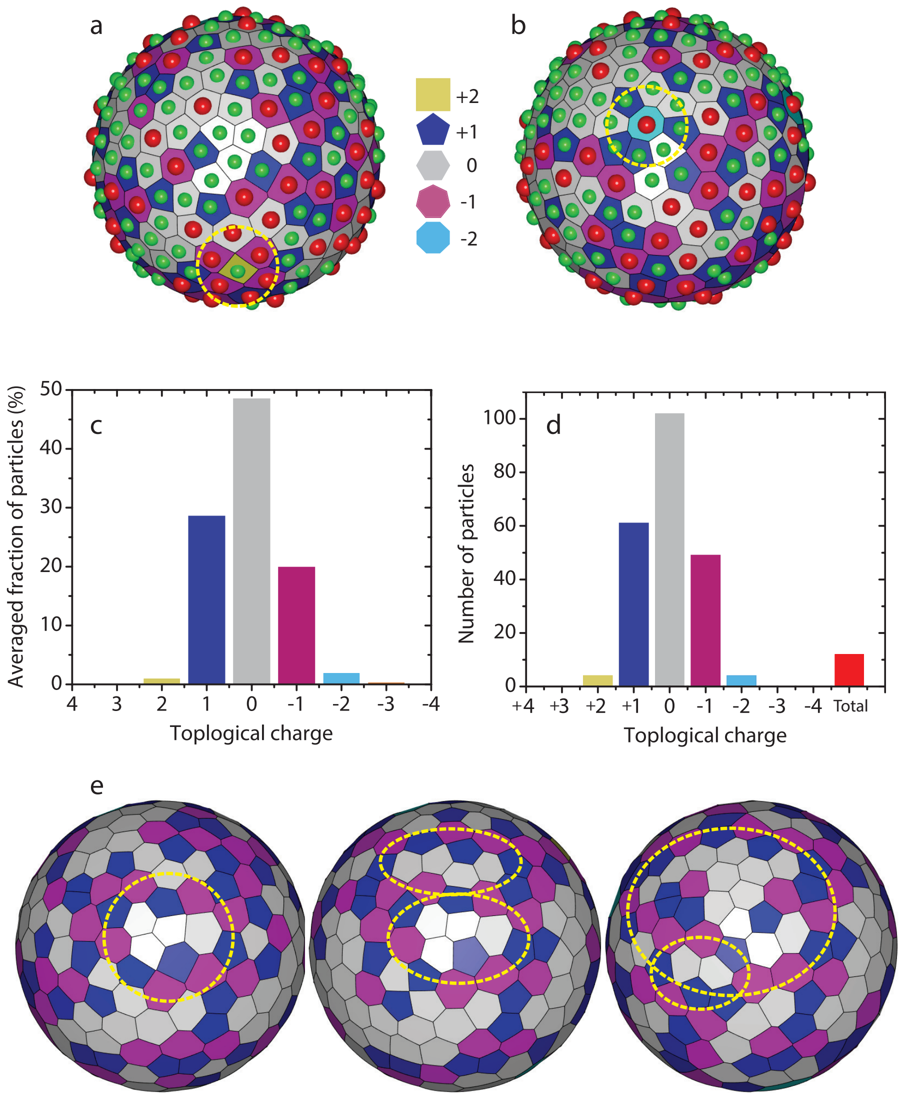

Supplementary Figure 2: **Statistics & morphology of topological defects. (a) & (b)** Dashed yellow circles show 4-fold (yellow rectangle, +2 topological charge) and 8-fold (cyan octagon, -2 topological charge) coordination, which occur occasionally. **(c)** Distribution of time averaged fraction of particles versus topological charge for $\Gamma = 63$. Here yellow, blue, gray, magenta, cyan and orange histograms are representing +2, +1, 0, -1, -2 and -3 topological charges or 4, 5, 6, 7, 8 and 9 fold coordination numbers, respectively. **(d)** Distribution of the instantaneous number of particles versus topological charge for $\Gamma = 63$ for a 3D stack. Here, the total topological charge 12 for a 3D stack is shown in red and all other colours are same as the previous plot. **(e)** Topological defects of bi-dispersed colloidal particles on the surface of a sphere show branching and closed-loop formations. The yellow dashed ellipses show different types of closed defect loops.

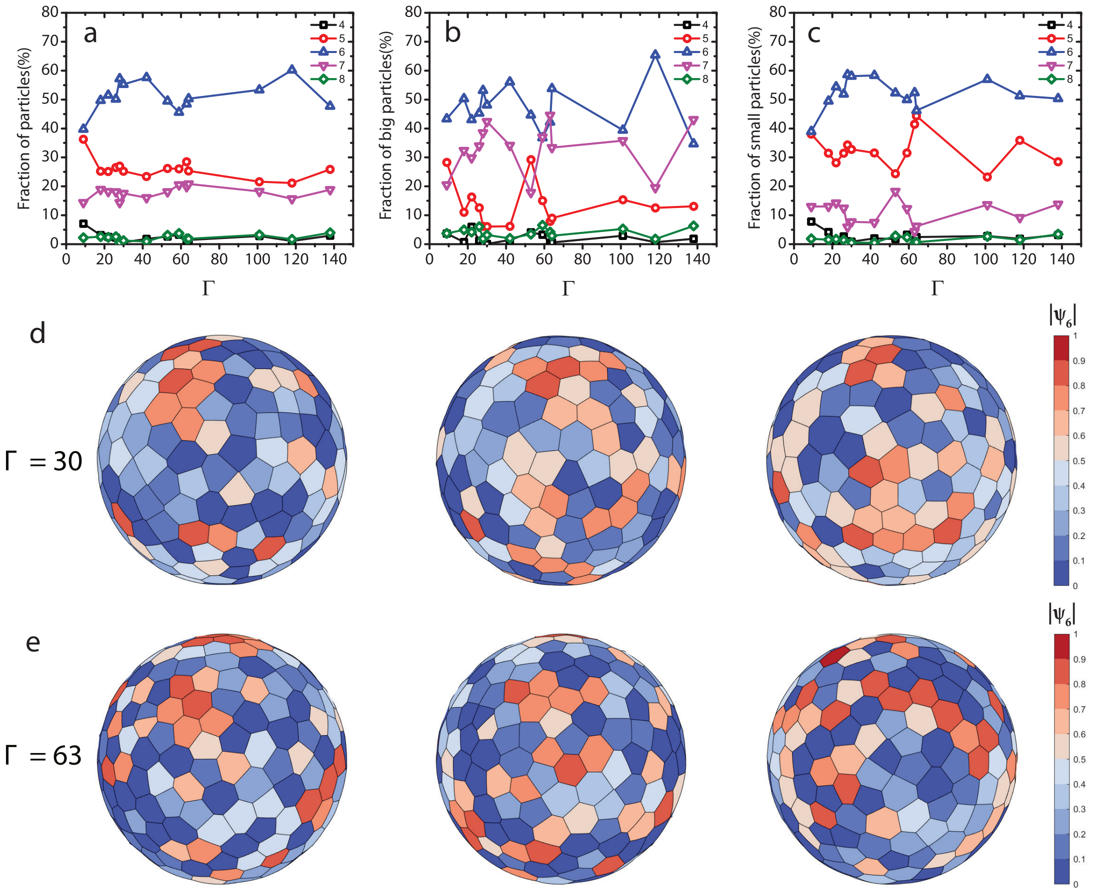

Supplementary Figure 3: **Coordination number as function of** Γ **and hexagonal bond-orientation order parameter** ψ_6 **at various times. (a)** The fraction of particles with a given coordination versus Γ averaged over the experiment duration. Here black, red, blue, magenta, and olive curves are associated with 4, 5, 6, 7, and 8 fold coordination numbers or correspondingly +2, +1, 0, -1, and -2 topological charge, respectively. **(b) & (c)** The fraction of big and small particles with a given coordination versus Γ averaged over the experiment duration, respectively. Here the colors are the same as in the previous plot. **(d) & (e)** Snapshots of hexagonal bond-orientation order parameter, $|\psi_6|$, on the surface of a sphere for $\Gamma = 30$ and $\Gamma = 63$, respectively, at three different time instances separated by $t > t^*$. These results clearly show the absence of crystalline order and also any demixing effects with time.

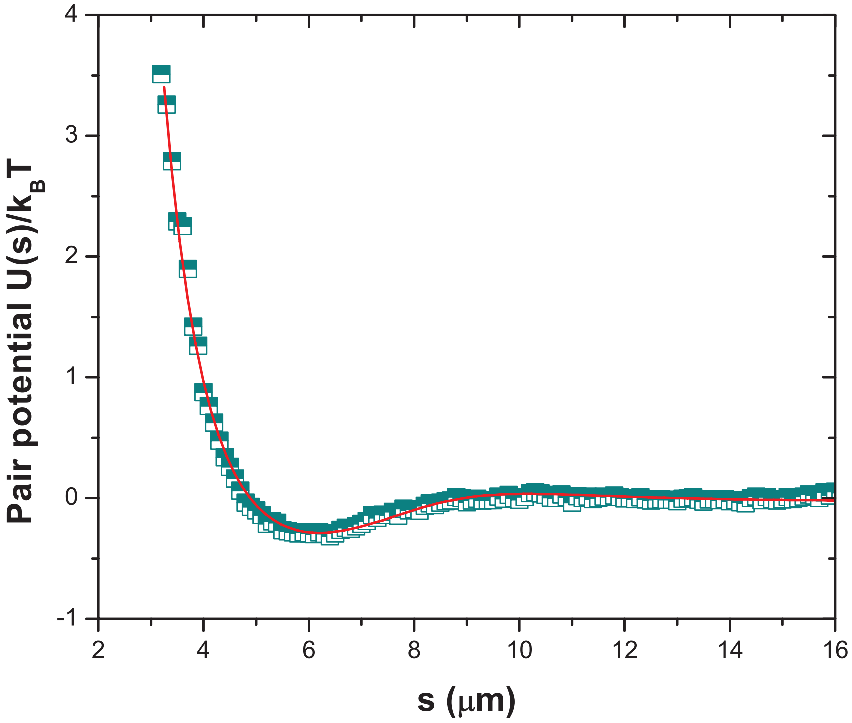

Supplementary Figure 4: **Calculating the particle pair interaction energy from experimental data.** The pair interaction energy $U(s)$ calculated from the pair correlation function $g(s)$ in the HNC approximation (dark gray squares) together with a fit to Supplementary Equation (10) (red line).

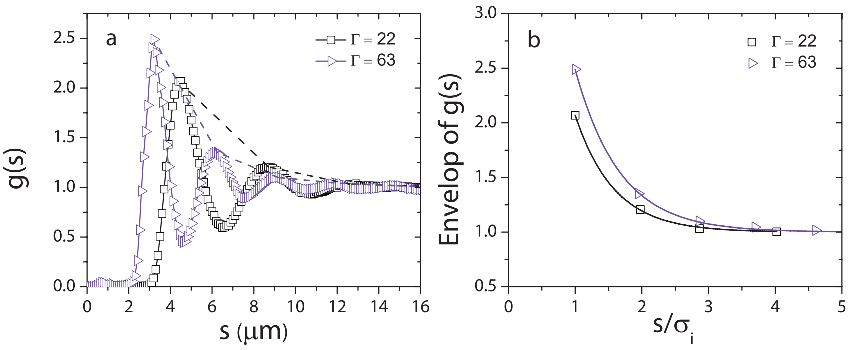

Supplementary Figure 5: **Decay of pair correlation function** $g(s)$ **& correlation length.** **(a)** Pair correlation function ***g(s)*** as a function of the geodesic distance ***s*** for $\Gamma = 22$ (black open squares) and $\Gamma = 63$ (violet open triangles). The dashed lines are the envelopes of maxima of $g(s)$. **(b)** The envelopes of maxima of $g(s)$ (symbol) and exponential fit to envelopes (lines) versus s/σ_i, where σ_i is the position of first peak of the corresponding *g(s)*. Fits for Γ =22 and $\Gamma = 63$ show exponential decay with correlation length $\leqslant 2\sigma_i$.

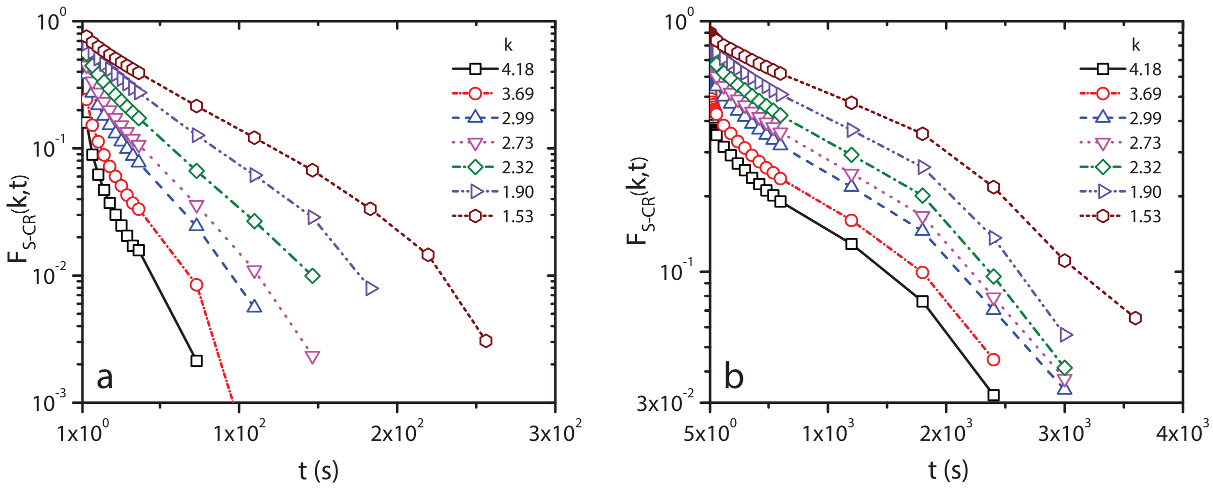

Supplementary Figure 6: **Self-intermediate scattering function** $F_{s-CR}(k,t)$**. (a) & (b)** Show a log-linear plot of $F_{s-CR}(k,t)$ versus t for various k values for $\Gamma = 30$ and $\Gamma = 63$, respectively.

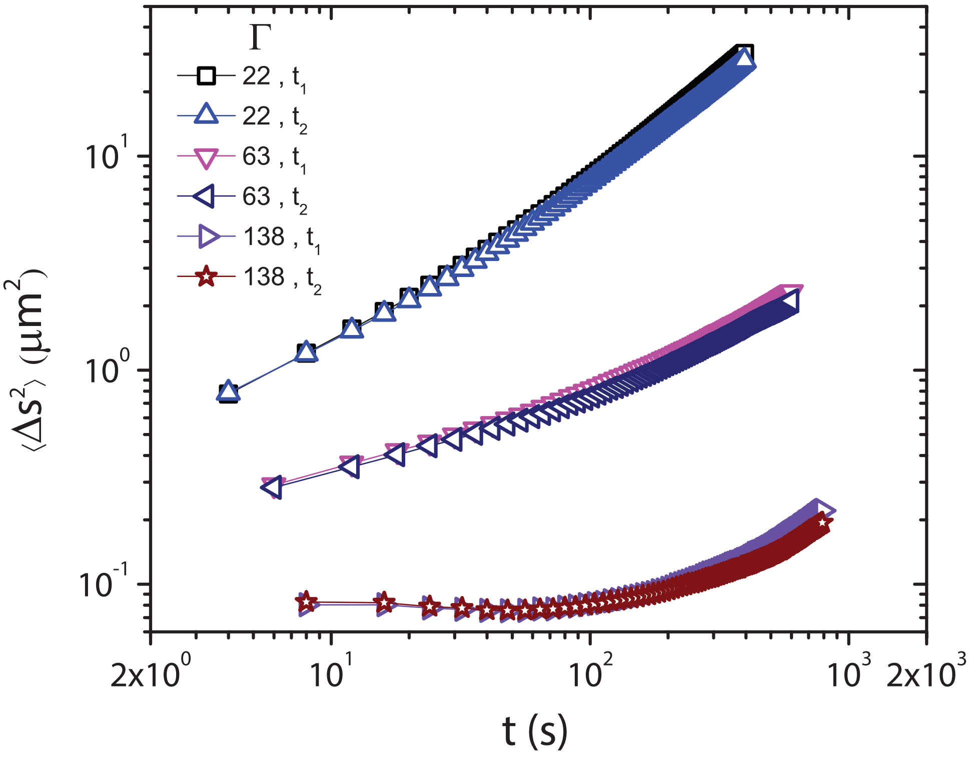

Supplementary Figure 7: **Probing aging effects** The mean-squared displacement $\langle \Delta s^2 \rangle$ versus t for $\Gamma = 22$, 63, & 138 over two time intervals t_1 and t_2. Here, t_1 and t_2 correspond to the first and last 100 3D-stacks of our experiments, respectively. The MSD over these two time windows are almost identical indicating aging effects are negligible in our experiments.

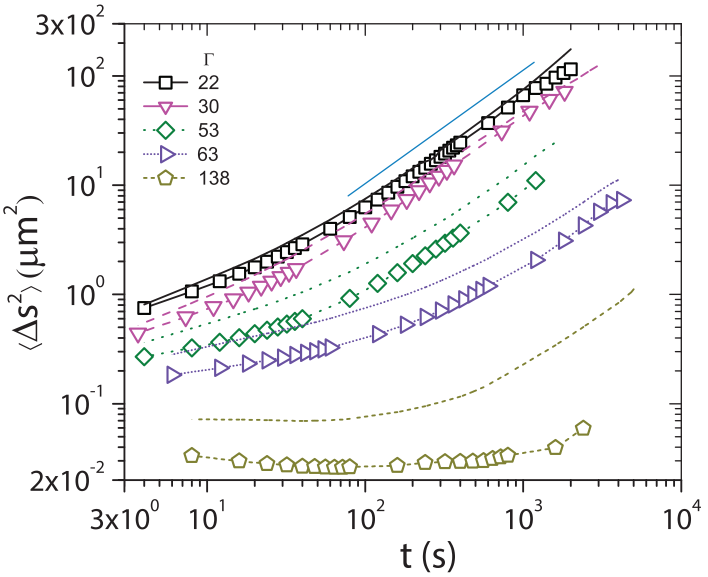

Supplementary Figure 8: **Mean-squared displacement on the surface of a sphere.** The conventional mean-squared displacement MSD (lines) and the cage-relative mean-squared displacement $MSD_{\rm CR}$ (symbols) for various Γ values. The conventional diffusion constant D and cage-relative diffusion constant $D_{\rm CR}$ were obtained from the long time diffusive regime of MSD and $MSD_{\rm CR}$, respectively.

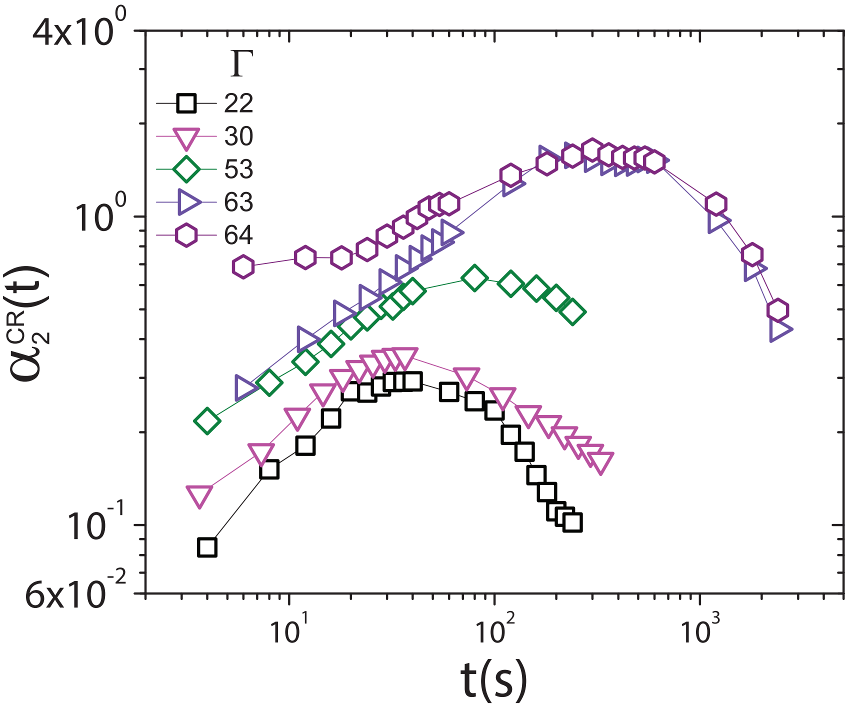

Supplementary Figure 9: **The cage-relative non-Gaussian parameter** $\alpha_2^{CR}(t)$ **on an** S^2 **sphere for various** Γ **values.** With increasing supercooling, the peak value of $\alpha_2^{CR}(t)$ increases, indicative of increasingly heterogeneous dynamics, and the location of the peak shifts to larger t indicative of dynamical slowing down.

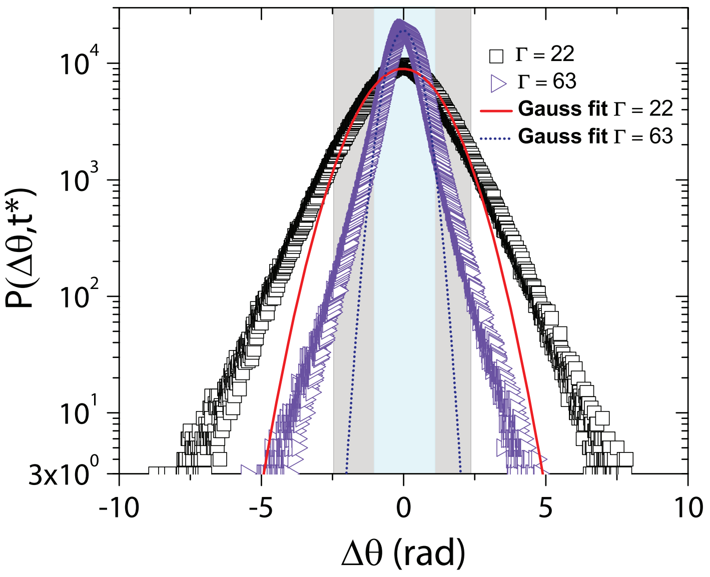

Supplementary Figure 10: **Dynamical heterogeneity.** The probability distribution of angular displacement $\Delta\theta$, over t^* (peak of the non-Gaussian parameter) for $\Gamma = 22$ (hollow black squares) and $\Gamma = 63$ (hollow violet triangle). The red solid and blue dotted lines are Gaussian fits to $P(\Delta\theta)$ for $\Gamma = 22$ and $\Gamma = 63$, respectively.

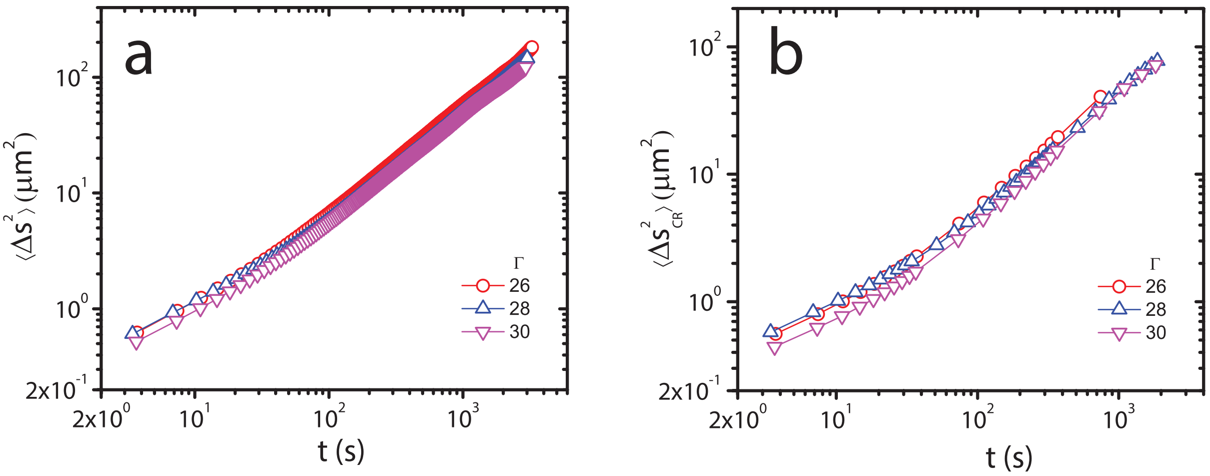

Supplementary Figure 11: **Dependence of mean-squared displacement** MSD **on number ratio of big and small particles.** **(a) & (b)** Show the conventional mean-squared displacement MSD and cage-relative mean-squared displacement MSD_{CR} versus Γ. For $\Gamma = 26$, 28, & 30, the number ratio $\left(\frac{M_b}{M_s}\right)$ is 1.1, 0.71, and 0.62, respectively, here M_b and M_s are the number of big and small particles. The nearly identical of MSD and MSD_{CR} profiles for different number ratio indicates that Γ characterizes the charged bi-dispersed system accurately on the surface of S^2 sphere.

Supplementary Note 1 | Rotational and translational drift correction

Experiments on emulsion droplets are known to have rotational and translation drifts due to various experimental factors such as objective lens motion in z-direction, stage drift and also rigid body rotation of the oil droplet. To remove these artifacts from the dynamics of colloidal particles, we calculated the rotational (rotation matrix about a different axis for each 3D image) and translational drift using the least-square error minimization and singular value decomposition (SVD) method of matrices [1]. The summary of the method for numerical computation is as follows.

Let us say, a set of M points $\boldsymbol{p} = (\boldsymbol{p_1}, \boldsymbol{p_2}, ..., \boldsymbol{p_M})$ at time t, transforms via a rotation matrix R and translation vector $\boldsymbol{T}$ to a set of points $\boldsymbol{q} = (\boldsymbol{q_1}, \boldsymbol{q_2}, ..., \boldsymbol{q_M})$ at time $t+1$ with an error $(\boldsymbol{q_i} - R\boldsymbol{p_i} - \boldsymbol{T})$. We have minimized the average square of the error $E(R, \boldsymbol{T})$.

$$E(R, \boldsymbol{T}) = \frac{1}{M} \sum_{i=1}^{M} ||(\boldsymbol{q_i} - R\boldsymbol{p_i} - \boldsymbol{T})||^2 \tag{1}$$

The steps to compute and minimize the rotation matrix R and translation vector $\boldsymbol{T}$ are given below.

1. Center-of-mass computation of both point sets $\boldsymbol{p}$ & $\boldsymbol{q}$:

$$\begin{aligned} \boldsymbol{\mu_q} &= \frac{1}{M} \sum_{i=1}^{M} \boldsymbol{q_i} \\ \boldsymbol{\mu_p} &= \frac{1}{M} \sum_{i=1}^{M} \boldsymbol{p_i} \end{aligned} \tag{2}$$

2. Position vectors in the center of mass frame:

$$\begin{aligned} \boldsymbol{y_i} &= \boldsymbol{q_i} - \boldsymbol{\mu_q} \\ \boldsymbol{x_i} &= \boldsymbol{p_i} - \boldsymbol{\mu_p} \end{aligned} \tag{3}$$

3. Computation of covariance matrix:

$$W = XY^{'} \tag{4}$$

where X & Y are $3 \times M$ matrices that have x_i and y_i as their columns, respectively and $Y^{'}$ is the transpose of Y.

4. To compute rotation matrix R we have performed singular value decomposition of $W = U\Sigma V^{'}$:

$$R = V \begin{pmatrix} 1 & & \\ & 1 & \\ & & det(VU^{'}) \end{pmatrix} U^{'} \tag{5}$$

5. Computation of optimal translation vector $\boldsymbol{T}$:

$$\boldsymbol{T} = \boldsymbol{\mu_q} - R\boldsymbol{\mu_q} \tag{6}$$

Supplementary Note 2 | Pair-correlation function $g(s)$ on a sphere

We calculated the pair correlation function $g(s)$ on the surface of a sphere as a function of geodesic distance s as:

$$g(s) = \frac{\int_\Omega dx \int_\Omega dx' \rho(x)\rho(x')\delta\left(|x-x'|-s\right)}{\left(M(M-1)V_\Omega^{-2}\right)\int_\Omega dx \int_\Omega dx' \delta\left(|x-x'|-s\right)} \tag{7}$$

where Ω is the surface of the sphere, M is the total number of particles. $|x-x'|$ is the geodesic distance between x and x', $\rho(x) = \sum_j \delta\left(r_j - x\right)$, r_j is the position of jth particle, and V_Ω is surface area of sphere.

Supplementary Note 3 | Hypernetted chain approximation on a sphere and calculation of interaction parameter Γ

In the limit of infinite dilution, the Boltzmann distribution relates the pair interaction energy $U(r)$ to the pair correlation function $g(r)$ as $\lim_{n\to 0} g(r) = \exp(-U(r)/k_BT)$, where n is the areal density of particles. At finite density and for soft particles, as is the case here, the relevant closure approximation is the hypernetted chain (HNC) approximation [2]. The pair interaction potential $U(r)$ can be calculated in the HNC approximation as

$$\frac{U(r)}{k_BT} = -\ln(g(r)) + nI(r) \tag{8}$$

where n is the areal density and $I(r) = \int [g(r') - 1 - nI(r)][g|r'-r| - 1]d^2r$.

We have extended HNC approximation to curved space by replacing radial distance r with the geodesic distance s.

$$\frac{U(s)}{k_BT} = -\ln(g(s)) + nI(s) \tag{9}$$

For charged colloidal particles at oil-water interface, Parolini et al. [3] have shown that the pair-interaction potential between particles at low densities can be parametrized as $\frac{U(s)}{k_BT} = \frac{A}{s^3}$. Whereas at higher density, the pair potential develops a minimum and the phenomenological parametrisation becomes:

$$\frac{U(s)}{k_BT} = \frac{A}{s^3} - \frac{B}{s^2} + \frac{\alpha}{\exp[\gamma(s_0 - s)] + 1}\frac{1}{s^2} \tag{10}$$

where A, B, α , γ and s_0 are fitting parameters. Here the first term $\frac{A}{s^3}$ in Supplementary Equation (10) generates dipolar behaviour at small s, the second term $\frac{B}{s^2}$ produces a minimum in the potential and the third term $\frac{\alpha}{\exp[\gamma(s_0-s)]+1}\frac{1}{s^2}$ ensures that the pair potential decays rapidly to zero at long distances [3].

From the HNC fits to the $g(s)$, we computed the particle pair-potential $U(s)$ for big

and small particles (Supplementary Figure 4). From the fits to $U(s)$, we determined the electric dipole moments for the big and small particles, A_b and A_s, respectively. Following previous experiments of charged bi-disperse colloids [4, 5], we defined a single dimensionless interaction parameter Γ, the ratio of electrostatic potential to the thermal energy, to characterize the system.

$$\Gamma = \frac{(\pi n)^{3/2}}{8\pi\epsilon k_B T}(\xi_b p_b + (1-\xi_b)p_s)^2 \tag{11}$$

where n is the areal density, ϵ is the dielectric constant, ξ_b is the fraction of big particles $\left(\frac{M_b}{M}\right)$, $\frac{p_b^2}{8\pi\epsilon} = A_b$ and $\frac{p_s^2}{8\pi\epsilon} = A_s$. Here, the total number of particles $M = M_b + M_s$ where M_b and M_s are the number of big and small particles on the surface.

Supplementary Note 4 | Self-intermediate scattering function $F_s(k,t)$ on a sphere

The Euclidean space self-intermediate scattering function $F_s(k,t) = \frac{1}{M}\sum_{j=1}^{M}\langle \exp(ik\Delta r_j(0,t))\rangle$ is appropriately modified on the surface of a S^2 sphere and is given by [6]:

$$F_s(k,t) = \frac{1}{M}\sum_{j=1}^{M}\left\langle P_{kR}\left(\cos\left(\frac{\Delta s_j(0,t)}{R}\right)\right)\right\rangle \tag{12}$$

Here, k is the wavevector corresponding to the first peak of $g(s)$, R is the radius of sphere, P_{n} is the nth Legendre polynomial and $\Delta r_j(0,t)$ and $\Delta s_j(0,t)$ are Euclidean and geodesic displacement of particle j over time t, respectively. To calculate the Legendre polynomials, kR was rounded off to the nearest integer.

Cage-relative measures are defined as:

$$\Delta s_{j-CR}(t) = \Delta s_j(0,t) - \frac{1}{NN_j}\sum_{i=1}^{NN_j}(s_i(t) - s_i(0)) \tag{13}$$

where, NN_j is number of nearest-neighbours of particle j and $\frac{1}{NN_j}\sum_{i=1}^{NN_j}(s_i(t) - s_i(0))$ is the geodesic displacement of centroid of the cage for the jth particle over time t.
Cage-relative self-intermediate scattering function $F_{s-CR}(k,t)$ is defined as:

$$F_{s-CR}(k,t) = \frac{1}{M}\sum_{j=1}^{M}\left\langle P_{kR}\left(\cos\left(\frac{\Delta s_{j-CR}(0,t)}{R}\right)\right)\right\rangle \tag{14}$$

In Fig. 2a of the main manuscript we have shown the Γ-dependence of $F_{s-CR}(k,t)$ at a fixed k. In Supplementary Figure 6, we show the k-dependence of $F_{s-CR}(k,t)$ for two different values of Γ.

Supplementary Note 5 | Mean-squared displacement (MSD) and non-Gaussian parameter $\alpha_2(t)$ on a S^2 sphere

Like the self-intermediate scattering function $F_s(k,t)$, the mean-squared displacement MSD is also modified on the surface of S^2 sphere and is defined as follows [7]:

$$MSD = -2R^2 \log\left(\left\langle \cos\left(\frac{|\Delta s_i(0,t)|}{R}\right)\right\rangle\right) \tag{15}$$

Similarly, the cage-relative mean-squared displacement MSD_{CR} is defined as:

$$MSD_{\mathrm{CR}} = -2R^2 \log\left(\left\langle \cos\left(\frac{|\Delta s_{i-CR}(0,t)|}{R}\right)\right\rangle\right) \tag{16}$$

The conventional MSD and the cage-relative MSD as a function of Γ are shown in Supplementary Figure 8.

The non-Gaussian parameter $\alpha_2(t)$ is also modified on the surface of S^2 sphere and is defined as follows [7]:

$$\alpha_2(t) = \frac{\log\left(\langle P_2\left(\cos\left(|\Delta s_i(0,t)|/R\right)\right)\rangle\right) - 3\log\left(\langle\cos\left(|\Delta s_i(0,t)|/R\right)\rangle\right)}{3\log\left(\langle\cos\left(|\Delta s_i(0,t)|R\right)\right)\rangle)^2} \tag{17}$$

The corresponding cage-relative non-Gaussian parameter $\alpha_2^{CR}(t)$ on surface of S^2 sphere is defined as [7]:

$$\alpha_2^{CR}(t) = \frac{\log\left(\langle P_2\left(\cos\left(|\Delta s_{i-CR}(0,t)|/R\right)\right)\rangle\right) - 3\log\left(\langle\cos\left(|\Delta s_{i-CR}(0,t)|/R\right)\rangle\right)}{3\log\left(\langle\cos\left(|\Delta s_{i-CR}(0,t)|R\right)\right)\rangle)^2} \tag{18}$$

Supplementary Note 5.1 | Dependence of MSD on number ratio of big and small particles

In our experiments, manual shaking results in emulsion droplets with varying number ratios $\left(\frac{M_b}{M_s}\right)$ of big and small particles. We have checked the effect of varying number ratio on dynamical quantities like mean-squared displacement MSD and cage-relative mean-squared displacement MSD_{CR} (Supplementary Figure 11). We have plotted MSD and MSD_{CR} for Γ = 26, 28, & 30, with number ratios 1.1, 0.71, and 0.62, respectively. In Supplementary Figure 11, for nearby Γ = 26, 28, & 30, the MSD and MSD_{CR} are similar although the number ratios are far apart (1.1, 0.71, & 0.62). This indicates that the interaction parameter Γ (Supplementary Equation (11)), which accounts for the fraction of big and small particles, nicely captures the dynamics of the charged bi-dispersed system on the surface of S^2 sphere.

Supplementary References

[1] Besl, P. J. & McKay, N. D. A method for registration of 3-d shapes. *IEEE Trans. Pattern Anal. Mach. Intell.* **14**, 239–256 (1992).

[2] Behrens, S. H. & Grier, D. G. Pair interaction of charged colloidal spheres near a charged wall. *Phys. Rev. E* **64**, 050401 (2001).

[3] Parolini, L., Law, A. D., Maestro, A., Buzza, D. M. A. & Cicuta, P. Interaction between colloidal particles on an oil–water interface in dilute and dense phases. *J. Phys.: Condens. Matter* **27**, 194119 (2015).

[4] Ebert, F., Dillmann, P., Maret, G. & Keim, P. The experimental realization of a two-dimensional colloidal model system. *Rev. Sci. Instrum.* **80**, 083902 (2009).

[5] Vivek, S., Kelleher, C. P., Chaikin, P. M. & Weeks, E. R. Long-wavelength fluctuations and the glass transition in two dimensions and three dimensions. *Proc. Natl Acad. Sci. USA* **114**, 1850–1855 (2017).

[6] Tarjus, G., Sausset, E. & Viot, P. Statistical mechanics of liquids and fluids in curved space. *Adv. Chem. Phys.* **148**, 251 (2012).

[7] Guerra, R. E., Kelleher, C. P., Hollingsworth, A. D. & Chaikin, P. M. Freezing on a sphere. *Nature* **554**, 346–350 (2018).
