# Supplementary material for: Cooperatively rearranging regions change shape near the mode-coupling crossover for colloidal liquids on a sphere
Source: Nat Commun. 2020 Oct 2;11:4967. doi: 10.1038/s41467-020-18760-7 (PMC7532192; doi:10.1038/s41467-020-18760-7)
Supplement: Supplementary file 3 — Description of Additional Supplementary Files [file 41467_2020_18760_MOESM3_ESM.pdf]

Description of Additional Supplementary Files

Supplementary Movie 1

Three-dimensional view of bi-dispersed colloidal particles wrapped on the surface of the S^2 sphere for $\Gamma = 63$.

Supplementary Movie 2

Particle trajectories for $\Gamma = 22$ and $\Gamma = 63$ on the surface of the S^2 sphere. Particles show diffusive motion at $\Gamma = 22$. Whereas for $\Gamma = 63$, particles remain caged for a long time.

Supplementary Movie 3

Particle displacement map over the cage-breaking time t^* for $\Gamma = 22$. Particles are colored according to their overlap values with red representing high particle overlaps over t^* and blue representing poor overlaps. Here, a poor overlap is an indication of a particle hopping out of its cage.

Supplementary Movie 4

Particle displacement map over the cage-breaking time t^* for $\Gamma = 63$. The system is dynamically heterogeneous behavior and rearrangements are cooperative. Here the color maps are same as in **Supplementary Movie 3**.

Supplementary Movie 5

Particle trajectories on the surface of an S^2 sphere show intermittent caging with abrupt hops, behavior typical of deeply supercooled liquids in 3D-Euclidean space.
