# Supplementary material for: Cooperatively rearranging regions change shape near the mode-coupling crossover for colloidal liquids on a sphere
Source: Nat Commun. 2020 Oct 2;11:4967. doi: 10.1038/s41467-020-18760-7 (PMC7532192; doi:10.1038/s41467-020-18760-7)
Supplement: Supplementary file 2 — Peer Review File [file 41467_2020_18760_MOESM2_ESM.pdf]

Reviewers' comments:

Reviewer #1 (Remarks to the Author):

In this manuscript the authors present the results on the relaxation dynamics of a glass-forming system that is confined on the surface of a sphere. This work is original since there are only few investigations in which such a topology has been considered and this setup does indeed allow to probe whether some of the theoretical approaches used to describe the slowing down of the dynamics in glass-forming systems are reasonable or not. The presented results are interesting and allow to advance the field of glass physics. However at present these results are not presented in a convincing manner and therefore the manuscript should be improved (see below for details). Once the various points have been addressed in a satisfactory manner I'm happy to recommend this manuscript to be accepted.

Specific points:

Page 3, top: "...stem from the inability of the locally favored structure - an icosahedron.." The relevance of icosahedral structures might be correct for one-component systems, but for multi-component systems this is not true. So the authors should be more precise on this point.

Page 3: "Since MCT is a mean-field theory, the singularity..." It would be useful if the authors would be more specific what singularity they talk about.

Page 4: "Gamma, which is the ratio of neighboring electrostatic dipole and thermal energies" The way this is formulated it seems as if the particle density plays no role. I have a hard time to believe this.

Page 5: "R = 19.2 ± 1.2r" I suggest to write "R = (19.2 ± 1.2)r" to make things clearer.

Page 5: "preferred coordination in 2D-flat space is six" This is only true for a one-component system. So the authors should be more precises.

Page 5: "spatial curvature and particle size bidispersity result in disclinations" The concept of "disclination" is useful for a crystalline system. For a liquid it makes no sense. The authors should rephrase this.

In the context of Fig. 1: How does the coordination number behave as a function of Gamma? Perhaps it is also useful to look at the partial coordination numbers. One has to make sure that the non-monotonic behavior seen in the structure (Fig 3b) is not related to some sort of trivial local demixing effect. By watching the movies one has the impression that some regions are relatively well ordered, i.e. that there are crystallites. How did the authors check for the presence of such ordered structures? The g(r) is usually a bad indicator to see such order. Perhaps look at the distribution of the bond angles?

Page 6: Here the authors introduce the self intermediate scattering function generalized to the curved space. This looks sophisticated, but at the end this observable is not that telling. I think it is more useful to look directly at the mean squared displacement (discussed in the SI) and at the self overlap function. The definition of the latter includes a parameter (the width of the function) that can be used as a proxy for the wave-vector used in the Fs(k,t).

Figure 2 shows the Fs-CR(k,t) as a function of time. Since for the highest Gamma this function does not decay to zero one starts to wonder whether the sample is indeed equilibrated. I didn't not find any information in the manuscript about the equilibration time. Did I miss it? Anyway, it is of utmost importance that the authors demonstrate that their results are not affected by aging/out of equilibrium dynamics. For the moment this question is not addressed at all. Note that the non-monotonic behavior of certain data, here a used to make a case in favor of RFOT, can very well be just an effect of the system falling out of equilibrium.

Page 7: "substantial contribution to the decay of Fs(k,t) stems from processes that do not contribute to structural relaxation." This makes no sense since Fs(k,t) measures structural relaxation. I guess what the authors want to say is that the local connectivity between the particles does not change.

Fig. 2a: At what time does the beta-relaxation start? Can the authors give an estimate?

Fig 2b: It would be useful to have beta for Gamma=40 so that the gap in the data can be closed.

Page 7, middle: Why use Gamma_g instead of Gamma_MCT? The "g" is for "glass"? We don't have a glass yet, just a viscous fluid. NB: In the caption of Fig3 the authors refer to Gamma_MCT.

Page 7: It would be useful to show alpha_2(t) for the different Gamma so that one sees better how the dynamical heterogeneities look like. (Cna be put in the SI.)

Page 7: The authors refer to "overlap" without giving any definition of that quantity. Please do so.

Fig 3a: These graphs look very homogeneous to my eye. It is not evident at all that there is a significant dynamical heterogeneity.

Page 8: "<Nc> has been found to decrease beyond ph_g in hard particle liquids and is attributed to cooperative dynamics associated with the short-time beta-relaxation process" I don't agree with this. Short time beta is the time window in which the correlation function *approaches* the plateau at intermediate time scales. There is no reason why this time scale should influence Nc, a quantity that is related to the alpha-process.

Page 8: "the compact core begins to dominate their overall morphology." I don't think so. RFOT predicts that at the MCT transition the length of the linear excitations becomes very large and beyond phi_MCT this length decreases again.

Page 8: "and the distribution also becomes broader" (Fig 3c). From the data it is not evident at all that the distribution becomes broader.

Page 8: The paper ends with a lengthy discussion on the Mermin-Wagner fluctuations (and this idea is presented already at several places before). But at the end it is concluded the these fluctuations are not relevant, and this is indeed not surprising since the system is rather small (order 20-30 particle diameters). So I'm not sure that it is useful to put forward a longish discussion and at the end to conclude that the effect is irrelevant. Why not put this in the SI and to free space for showing the reader the static and dynamic quantities that are relevant for the glassy behavior of the system?

Figure 4: It is remarkable that the Stokes-Einstein relation doe snot even hold at low Gamma. Any comments by the authors on this surprising result?

SI:
Regarding the system: If the PMMA particles pick up the charges, are these all the same? In other words, the fact that one has large and small particles does this mean that one has indeed only two species or does one have two *distributions* that need to be taken into account for obtaining the effective potential?

SI-Fig5: I find it very surprising that all functions seem to go to zero at the same time. Is this really reasonable? Why?

Eq. 16: It would be more appropriate to calculate the MSD by integrating the displacements of the particles between the different video frames.

Movies:
Movie1: One has the impression that locally the system tends to phase separate. True or not?

Movie2: For Gamma=63 there are instances in which the particles sit on top of each other. How is this possible?

Movie3: I think that there are too many red patches: Are the authors sure to have made a good choice of t*?

The conclusion should be strengthened: The authors write that MCT/RFOT is able to rationalize these results. Does this really imply that the competing theories can be discarded? A more explicit statement by the authors on this question would be welcome.

Reviewer #2 (Remarks to the Author):

I am very much pleased by this manuscript.
It is not very common to read an experimental study explained in
such a clear way. It is also not very common to read a manuscript focusing on fundamental themes associated to the glass transition based on clean experimental data.

The authors present a quite comprehensive study of the slowing down of the dynamics on increasing the particle concentration, when particles are trapped on a spherical water-oil interface. The authors interrogate their data to extract information on:

1) The interaction potential
2) The absence of crystallinity
3) The role of the cage dynamics
4) The connection between interaction strength and dynamic properties
5) The spacial structure of the CCR
6) The importance of anomalous dynamics effects related to the behavior of the density of states in two dimensions (Mermin-Wagner, MW)

All these issues are properly presented, addressed, discussed, commented.

The most important results steaming out from this experimental study are

1) The demonstration that dynamics follow a MCT-like dependence
2) That the morphology of the CRR near the MCT cross-over changes from linear to compact
3) That MW-fluctuations do not spoil the results in the limit of strong curvature

These results are relevant, since they provide support for a description of the glass dynamics based on random first order theory, a theory that extends MCT to account for activate processes below the MCT cross-over. It is also
worth noticing that this is the first time that dynamics on a sphere is experimentally investigated, opening the possibility of careful comparison with numerical and theoretical predictions.

I have only very minor suggestions to propose to improve this manuscript.

1) Discuss in the main text the reasons why random rotation of the oil droplet (as a rigid body) can be neglected
2) Discuss the possibility that the "network" of particles on the sphere surface undergoes a Brownian rotational process as a whole, a spurious effect which perhaps could be as relevant as the MW fluctuations.
3) Clarify the notation P_{kR}. I was expecting to see an integer in the subscript of P. kR can be real. Also, a reference defining the self-intermediate scattering function on a sphere could be useful.
4) .. as predicted by MCT. Perhaps better
... a value consistent with MCT predictions.
5) ...had MW fluctuations.... perhaps better .... had significant MW fluctuations...

Reviewer #3 (Remarks to the Author):

Singh, Sood, and Ganapathy examined a bi-disperse colloidal liquid trapped on the surface of a sphere. This is an experimental realization of a two-dimensional glassy system. The curvature and the finite size of the system gives it characteristics that are different than for two-dimensional and three-dimensional systems. Two of the author's conclusions are that the mode-coupling theory describes the dynamics and that the cooperatively rearranging regions change from string-like to compact near the mode-coupling crossover. Additionally, the Mermin-Wagner fluctuations that have been determined to be very important for two-dimensional liquids are irrelevant for liquids on a sphere.

The experiment is interesting and the analysis is well done. I have some questions about comments about the discussions and conclusions that need to be addressed before I can recommend the paper for publication.

1) The non-monotonic evolution of the height of the first peak of of the pair correlation function is only due to one point. The authors should add another point at for Gamma greater than 80 to verify this non-monotonic evolution.

2) In Figure 2a, the cage relative is shorter for Gamma = 9 than the non-cage relative relaxation time, but the non-cage relative relaxation time is shorter at every other Gamma. Can the authors explain why Gamma = 9 is different?

3) Figure 2a is hard to follow at first. By connecting the symbols with lines it takes a reader awhile to understand the figure.

4) As a comment, it is unsurprising that the mode-coupling-theory provides a good fit to the first three decades of slowing down observed in these experiments since this is observed in many liquids. One generally needs several more decades of slowing down to observe deviations from the mode-coupling-theory. Therefore, I don't find this observation to warrant publication in Nature Communications.

5) This is not the first time it has been reported that the shapes of cooperatively re-arranging regions are changing from string-like to compact near the mode-coupling crossover. This change in shape was shown in the work of Flenner, Staley, and Szamel (PRL 112, 097801, 2014). Other authors have inferred a change of shape also. These previous works should be discussed in the manuscript.

Due to the issues listed above, I do not think the authors have demonstrated that the results are novel enough for publication in Nature Communications.

Response to Reviewers' comments:

Reviewer #1 (Remarks to the Author):

Comment: *In this manuscript the authors present the results on the relaxation dynamics of a glass-forming system that is confined on the surface of a sphere. This work is original since there are only few investigations in which such a topology has been considered and this setup does indeed allow to probe whether some of the theoretical approaches used to describe the slowing down of the dynamics in glass-forming systems are reasonable or not. The presented results are interesting and allow to advance the field of glass physics. However, at present these results are not presented in a convincing manner and therefore the manuscript should be improved (see below for details). Once the various points have been addressed in a satisfactory manner, I'm happy to recommend this manuscript to be accepted.*

Response: We thank the reviewer for appreciating the salient features of our study and for finding our results interesting and helping advance the field of glass physics. We thank them for their critical comments which has helped enhance our manuscript. We address below the points raised by the referee.

Specific points:

Comment 1: *Page 3, top: "...stem from the inability of the locally favored structure - an icosahedron." The relevance of icosahedral structures might be correct for one-component systems, but for multi-component systems this is not true. So, the authors should be more precise on this point.*

Response 1: In a recent numerical study, Turci and coworkers (**Ref. [7] of the Main Manuscript, Line No: 4-7**) showed that frustration in a Wahnström mixture, a binary LJ mixture with particle size ratio of 6/5, is relieved when 3D-Euclidean space is curved into a 4-dimensional hypersphere (also called the 3-sphere). On the 3-sphere, for N= 120 particles, an icosahedral ordering - {3,3,5} polytope - was observed in this study. Thus, even when particle bidispersity is present, curving space in higher-dimension can potentially relieve frustration and this is what we have alluded to.

Comment 2: *Page 3: "Since MCT is a mean-field theory, the singularity..." It would be useful if the authors would be more specific what singularity they talk about.*

Response 2: We thank the referee for the comment. We are now more precise about what singularity we refer to in the revision (**Line No: 23 of Main Manuscript**).

Comment 3*: Page 4: "Gamma, which is the ratio of neighboring electrostatic dipole and thermal energies" The way this is formulated it seems as if the particle density plays no role. I have a hard time to believe this.*

Response 3: We apologize for the lack of clarity as there was a typo. The referee is indeed correct. Γ is the ratio of the total electrostatic energy and thermal energy and hence depends on the number of particles on the surface of the sphere. We have corrected this in the revision and have also included the expression for Γ (**Line No: 63, 64-66 of Main Manuscript**).

Comment 4: *Page 5: "R = 19.2 ± 1.2r" I suggest to write "R = (19.2 ± 1.2)r" to make things clearer.*

Response 4: This has been corrected in the revision (**Line No: 70**).

Comment 5: *Page 5: "preferred coordination in 2D-flat space is six" This is only true for a one-component system. So, the authors should be more precise.*

Page 5: "spatial curvature and particle size bidispersity result in disclinations" The concept of "disclination" is useful for a crystalline system. For a liquid it makes no sense. The authors should rephrase this.

Response 5: We thank the referee for these comments and have now rectified this in our revision (**Line No: 73 and 75 of Main Manuscript**). Since disclinations are also used in the context of liquid crystals, we had used it. We now refer to these as coordination defects to avoid confusion.

Comment 6*: In the context of Fig. 1: How does the coordination number behave as a function of Gamma? Perhaps it is also useful to look at the partial coordination numbers. One has to make sure that the non-monotonic behavior seen in the structure (Fig 3b) is not related to some sort of trivial local demixing effect. By watching the movies, one has the impression that some regions are relatively well ordered, i.e. that there are crystallites. How did the authors check for the presence of such ordered structures? The g(r) is usually a bad indicator to see such order. Perhaps look at the distribution of the bond angles?*

Response 6: Following the referee's comments, we have now calculated both the coordination number and the partial coordination number as a function of Γ and this is shown in **Supplementary Fig. S3a-c.** The fraction of particles with a given coordination, both partial and otherwise, versus Γ fluctuates about the horizontal and is a clear signature that there is no demixing effect when Γ increases. We also checked for the presence of crystalline order by calculating the hexagonal bond-orientational order parameter $|\psi_6|$ at various time instances for two different values of Γ. This analysis (**Supplementary Fig. S3d,e**) did not reveal any crystalline ordering. Thus, the bidispersity used in our study is sufficient to thwart crystallization **(Line No: 87-89 of Main Manuscript**).

Comment 7: *Page 6: Here the authors introduce the self-intermediate scattering function generalized to the curved space. This looks sophisticated, but at the end this observable is not that telling. I think it is more useful to look directly at the mean squared displacement (discussed in the SI) and at the self-overlap function. The definition of the latter includes a parameter (the width of the function) that can be used as a proxy for the wave-vector used in the Fs(k,t).*

Response 7: We used the self-intermediate scattering function (ISF) to make contact with previous studies of liquids on curved surfaces, where this quantity is evaluated, and to carry of the MCT scaling analysis. This function is more complex for a sphere, but like in Euclidean space, allows the wave-vector dependence of the dynamics to be probed. Since this is the conventional manner for performing the MCT scaling analysis, we would like to retain it. If the referee strongly feels that these should be moved to the supplement, we will be happy to do so.

Comment 8: *Figure 2 shows the Fs-CR(k,t) as a function of time. Since for the highest Gamma this function does not decay to zero one starts to wonder whether the sample is indeed equilibrated. I didn't not find any information in the manuscript about the equilibration time. Did I miss it? Anyway, it is of utmost importance that the authors demonstrate that their results are not affected by aging/out of equilibrium dynamics. For the moment, this question is not addressed at all. Note that the non-monotonic behavior of certain data, here a used to make a case in favor of RFOT, can very well be just an effect of the system falling out of equilibrium.*

Response 8: We thank the referee for this comment. To determine if there was any aging of the samples, we calculated the MSD over the first and the last 100 frames (z-stacks) of the experiment. The MSD are almost identical over the entire range of Γ's studied and clearly shows there is no aging behaviour in these samples. These are shown in **Supplementary Fig. S7** and is also mentioned in **Line No: 134-135 of the Main Manuscript**. In our experiments, data collection starts within 20 minutes to 1-hr of sample loading. These samples are quite sensitive and longer wait times are not possible. Having said that, we would like to emphasize that the stability of our samples is comparable or exceeds those on a similar system reported recently (Nature 554, 346 (2018), **Ref. [4] of Manuscript**). To the best of our knowledge, ours is the first stable system of bidisperse particles on a sphere.

Comment 9: *Page 7: "substantial contribution to the decay of Fs(k,t) stems from processes that do not contribute to structural relaxation." This makes no sense since Fs(k,t) measures structural relaxation. I guess what the authors want to say is that the local connectivity between the particles does not change.*

Response 9: We have reworded this sentence in our revision (**Line No: 141 of Main Manuscript**).

Comment 10: *Fig. 2a: At what time does the beta-relaxation start? Can the authors give an estimate?*

Response 10: Since we perform 3D imaging of the entire droplet, our confocal scanner, despite being the current state-of-the-art, can still not capture the very short-time scale dynamics. We nevertheless tried to determine the β-relaxation time scale, but we were not successful as our temporal resolution appears to larger than that corresponding to the β-process.

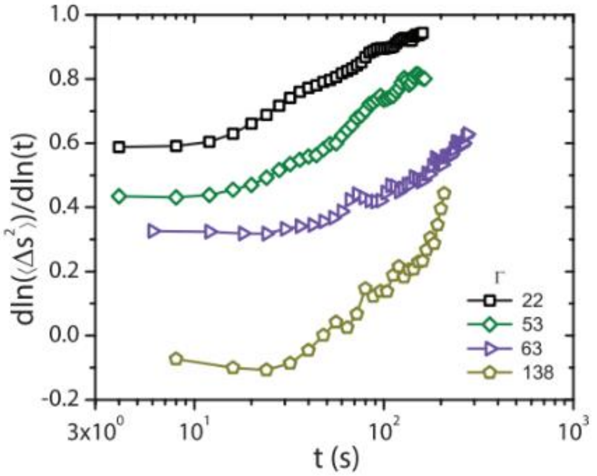

Fig 1: dln (MSD)/dln (t) versus t for various Γs. A minima in this plot at short times would denote the time scale of the β-process and is not accessible in our experiments.

***Comment 11**: Fig 2b: It would be useful to have beta for Gamma=40 so that the gap in the data can be closed.*

Response 11: We have now carried out two experiments, one for $\Gamma \sim 40$ and the other for $\Gamma \sim 120$ (asked by Referee 3) and the results of these experiments, combined with the previous ones, are shown in the **Inset to Fig. 1e, Fig 2b, Fig. 3c and Fig. 4 of the Main Manuscript**. These experiments only further reinforce our findings.

***Comment 12:** Page 7, middle: Why use Gamma_g instead of Gamma_MCT? The "g" is for "glass"? We don't have a glass yet, just a viscous fluid. NB: In the caption of Fig3 the authors refer to Gamma_MCT.*

Response 12: We agree with the referee and we now use Γ_{MCT} to denote the MCT singularity (**Line No: 149 and 155 of Main Manuscript**).

***Comment 13**: Page 7: It would be useful to show alpha_2(t) for the different Gamma so that one sees better how the dynamical heterogeneities look like. (Can be put in the SI.)*

Response 13: The cage-relative non-Gaussian parameter, $\alpha_{2\text{-}CR}(t)$, for various Γ values is now shown in **Supplementary Fig. S9**. The observed behaviour is typical of what is observed in Euclidean space with increasing supercooling.

***Comment 14:** Page 7: The authors refer to "overlap" without giving any definition of that quantity. Please do so.*

Response 14: We apologize for this oversight. We have corrected this in our revision (**Line No: 164 and 165**).

***Comment 15:** Fig 3a: These graphs look very homogeneous to my eye. It is not evident at all that there is a significant dynamical heterogeneity.*

Response 15: We apologize for the lack of clarity and we have now split Fig 3a into two figures that correspond to $\Gamma = 22$ **(Fig. 3a) and** $\Gamma = 63$ **(Fig. 3b)**, respectively. At low Γ, the dynamics is indeed homogenous, as is expected, whereas at high Γ, we see significant collective dynamics. This is also evident in Supplementary Movie 4.

***Comment 16**: Page 8: "<Nc> has been found to decrease beyond ph_g in hard particle liquids and is attributed to cooperative dynamics associated with the short-time beta-relaxation process" I don't agree with this. Short time beta is the time window in which the correlation function *approaches* the plateau at intermediate time scales. There is no reason why this time scale should influence Nc, a quantity that is related to the alpha-process.*

Response 16: We made this remark after being guided by the existing literature. In their seminal study on hard spheres, Weeks and coworkers found that $\langle N_c \rangle$ drops sharply beyond the glass transition and made a categoric remark that these clusters correspond to the β-process **Ref [36] of Main Manuscript**. Also, as seen by Weeks and coworkers, the value of $\langle N_c \rangle$ did not critically depend on the choice of t* in this regime of Γ. Such a drop in $\langle N_c \rangle$ beyond the MCT singularity has been observed in many other systems including liquids of colloidal ellipsoids. Further, in a recent numerical study, Karmarkar and

coworkers found that the temperature dependence of the cooperativity length scale associated with the β-process is same as the length scale associated with the α-process [PRL 116, 085701 (2016)]. They also suggest that short-time dynamics is possibly related to long-time structural relaxation. A correlation between the short-time and long-time relaxation has also been uncovered in recent colloid experiments by Arjun Yodh and coworkers (Mishra et al., PRE 100, 020603(R) 2019). On curved surfaces however, we do not yet know if these exact mechanisms are at work. We have now added a statement to this effect (**Line No: 181 of Main Manuscript**).

Comment 17: *Page 8: "the compact core begins to dominate their overall morphology." I don't think so. RFOT predicts that at the MCT transition the length of the linear excitations becomes very large and beyond phi_MCT this length decreases again.*

Response 17: Following the referee's suggestion, we have now reworded these lines (**Line No: 188-190 of Main Manuscript**).

Comment 18: *Page 8: "and the distribution also becomes broader" (Fig 3c). From the data it is not evident at all that the distribution becomes broader.*

Response 18: We thank the referee for this comment. Our statement was not accurate and the claim about P(NN) becoming broader has been removed in our revision (**Line No: 197**). We now only emphasize on the shift in the peak position with increasing Γ.

Comment 19: *Page 8: The paper ends with a lengthy discussion on the Mermin-Wagner fluctuations (and this idea is presented already at several places before). But at the end it is concluded these fluctuations are not relevant, and this is indeed not surprising since the system is rather small (order 20-30 particle diameters). So, I'm not sure that it is useful to put forward a longish discussion and at the end to conclude that the effect is irrelevant. Why not put this in the SI and to free space for showing the reader the static and dynamic quantities that are relevant for the glassy behavior of the system?*

Response 19: We have had a similar response from some of our theory colleagues. We would however like to point out that it is only in hindsight that our results do not seem surprising and can be attributed to a small system size. A priori it was not clear to us at all if the dynamics of liquids on a sphere would behave like liquids in Euclidean space, given that crystals in flat and curved space behave so differently. For the very same reason, we were also not sure if MW fluctuations, which are known to diminish in strength when system size is reduced, would not be significant for small spherical surfaces as well. How MW fluctuation grow as the sphere diameter is increased and whether its behaviour is same as the system size is increased in Euclidean space is worth exploring in future. Reviewer #2 was particularly pleased with these observations and we would therefore like to retain the discussions and results pertaining to MW fluctuations in the Main Manuscript. We hope the reviewer is aggregable with this.

Comment 20*: Figure 4: It is remarkable that the Stokes-Einstein relation does not even hold at low Gamma. Any comments by the authors on this surprising result?*

Response 20: We are unable to probe dynamics in the very low Γ regime since the dynamics is too fast to be captured by our confocal microscope in 3D imaging experiments. In 2D imaging of a slice of the 3D sphere, particles diffuse out of the imaging plane too rapidly and particle tracking was not possible. As the referee will notice, that even for $\Gamma = 22$, the MSD shows a transition from sub-diffusive dynamics at short-times to diffusion at long times and this is evident as a peak in $\alpha_{2\text{-}CR}(t)$ (**Supplementary Fig. S8**

and S9). In fact, even for $\Gamma = 9$, we had difficulties tracking particles due to fast dynamics. It is very much possible that the SE relation is not violated for Γ values smaller than lowest values accessible in our study. In 2D-Euclidean space, the violation of the SE relation at high temperatures or low densities has been attributed to MW fluctuations (Ref. [20] of Main Manuscript). Given that these do not seem be significant for the system size studied, the SE relation may be valid for very low values of Γ.

SI:

Comment 21: *Regarding the system: If the PMMA particles pick up the charges, are these all the same? In other words, the fact that one has large and small particles does this mean that one has indeed only two species or does one have two *distributions* that need to be taken into account for obtaining the effective potential?*

Response 21: The referee is very correct. PMMA particles of two different sizes do not pick up the same charge. We had indeed calculated the individual species pair-potentials by computing the partial pair-correlation function g(s), and then computing the U(s) in the HNC approximation. This is what was used in calculating Γ and is described in the **Supplementary Section 5**. At present, there are no clear strategies to ascertain if within each species, whether the charges are distributions, or they are sharply defined. Nevertheless, the fact that Γ so nicely parametrizes the system suggests that assuming the charges on each particle species to be sharply distributed is not an unreasonable assumption.

Comment 22: *SI-Fig5: I find it very surprising that all functions seem to go to zero at the same time. Is this really reasonable? Why?*

Response 22: We had by mistake represented the Figure in a linear-log plot. We have now rectified this in the revised supplement (**Supplementary Fig. S6**). With decreasing wavevector k, $F_{s\text{-}CR}(k,t)$ takes longer to decay, as is expected, and this is apparent in a log-linear representation. We thank the referee for pointing this out.

Comment 23: *Eq. 16: It would be more appropriate to calculate the MSD by integrating the displacements of the particles between the different video frames.*

Response 23: Unlike in numerical studies where particle positions are obtainable with very high accuracy, in experiments the spatial resolution with which particles can be tracked is finite (about 50 nm for a micron-sized colloid). When integrating particle displacements to calculate the MSDs, errors due to finite spatial resolution begin to accumulate and this makes the MSD calculated using this procedure unreliable at long-times.

Movies:

Comment 24*: Movie1: One has the impression that locally the system tends to phase separate. True or not?*

Response 24: A close inspection of the movies did not reveal any phase separation over the duration of our experiments. Any phase separation would have resulted in patches of particles with crystalline order and a hexagonal bond-order parameter analysis did not reveal any such ordering. The new analysis reported in **Supplementary Fig. S3d and e** support this observation.

Comment 25: *Movie2: For Gamma=63 there are instances in which the particles sit on top of each other. How is this possible?*

Response 25: We apologize for the lack of clarity in the movie and have now rectified it. The particles appearing to sit on top of each other is an artifact due to the front and back hemispheres represented in the same movie. We have now split the front and back hemispheres into two separate panels in the revised **Supplementary Movie 2**.

Comment 26: *Movie3: I think that there are too many red patches: Are the authors sure to have made a good choice of t*?*

Response 26: When the hemisphere is projected onto a plane for representation purposes, the particle density near the periphery appears larger. Most-mobile particle clusters appear more densely packed and also more in number near the periphery. In all our analysis, the choice of t* is from the peak of $\alpha_{2\text{-}CR}$(t) (**Supplementary Fig. S9**).

Comment 27*: The conclusion should be strengthened: The authors write that MCT/RFOT is able to rationalize these results. Does this really imply that the competing theories can be discarded? A more explicit statement by the authors on this question would be welcome.*

Response 27: In all studies probing glass physics on curved surfaces hitherto, all of them also numerical, the primary focus has been on frustration-based approaches and MCT. Our goal was to explore other glass theories as well and we found that the change in shape of CRRs, a prediction unique to RFOT, also applies on a sphere. This feature is currently not captured by other glass theories including kinetic approaches like Dynamical Facilitation. We have now mentioned this explicitly in the revision (**Line No: 230-231**). Being experimentalists, we do not know yet if there are clear routes to capturing our observations within these other theories. Until then any categoric statement about discarding other theories would be, in our view, inappropriate. Like we mentioned in our abstract, we are of the view that a comparative evaluation of key theoretical predictions from through critical experiments is much needed to uncover the true nature of the glass transition.

We hope the reviewer is satisfied with our responses and now finds the manuscript suitable for publication in Nature Communications.

Reviewer #2 (Remarks to the Author):

Comment: *I am very much pleased by this manuscript. It is not very common to read an experimental study explained in such a clear way. It is also not very common to read a manuscript focusing on fundamental themes associated to the glass transition based on clean experimental data.*

The authors present a quite comprehensive study of the slowing down of the dynamics on increasing the particle concentration when particles are trapped on a spherical water-oil interface. The authors interrogate their data to extract information on:

1) The interaction potential
2) The absence of crystallinity
3) The role of the cage dynamics
4) The connection between interaction strength and dynamic properties
5) The spatial structure of the CCR
6) The importance of anomalous dynamics effects related to the behavior of the density of states in two dimensions (Mermin-Wagner, MW)

All these issues are properly presented, addressed, discussed, commented.

The most important results steaming out from this experimental study are

1) The demonstration that dynamics follow a MCT-like dependence
2) That the morphology of the CRR near the MCT cross-over changes from linear to compact
3) That MW-fluctuations do not spoil the results in the limit of strong curvature

These results are relevant, since they provide support for a description of the glass dynamics based on random first order theory, a theory that extends MCT to account for activate processes below the MCT cross-over. It is also worth noticing that this is the first time that dynamics on a sphere is experimentally investigated, opening the possibility of careful comparison with numerical and theoretical predictions.

Response: We are extremely delighted that the referee liked our study and found it to be clear, relevant, and comprehensive. We also thank them for noting that these first experiments probing glassy physics on curved surfaces opens the possibility of comparing numerical/theoretical predictions. We have addressed the comments raised by the referee below.

I have only very minor suggestions to propose to improve this manuscript.

Comment 1: *Discuss in the main text the reasons why random rotation of the oil droplet (as a rigid body) can be neglected*

Response 1: We thank the referee for this comment. The rigid body rotation of the entire droplet cannot be neglected, and we have accounted for this when we dedrift the data. We had accidentally not included the reference to the Supplementary section, where these are discussed in detail. We have now corrected this in the revision (**Line No: 61 of Main Manuscript**). In our experiments, we did observe rotation of the droplets as a whole, over and above translational drift, and in **Supplementary Section 3**, we describe how this was corrected.

Comment 2*: Discuss the possibility that the "network" of particles on the sphere surface undergoes a Brownian rotational process as a whole, a spurious effect which perhaps could be as relevant as the MW fluctuations.*

Response 2: The sphere on which the particles reside is almost 30 μm in diameter and Brownian motion of the entire droplet can be safely neglected. The droplet as a whole can only translate and/or rotate due to small thermal gradients and/or the motion of the microscope objective during 3D imaging. This translational and rotational drift has been corrected prior to subsequent analysis and thus the network of particles on the surface is not subject to any artifacts.

Comment 3: *Clarify the notation P_{kR}. I was expecting to see an integer in the subscript of P. kR can be real. Also, a reference defining the self-intermediate scattering function on a sphere could be useful.*

Response 3: We thank the referee for this comment. *kR* is indeed rounded of to the nearest integer and this is now mentioned in the both the **Main Manuscript (Line No: 120-121)and Supplementary Section 6**. We have also cited the paper where this definition is introduced.

Comment 4: *.. as predicted by MCT. Perhaps better*
... a value consistent with MCT predictions.

Response 4: We have now rectified this in our revision (**Line No: 153**).

Comment 5*: ...had MW fluctuations.... perhaps better .... had significant MW fluctuations...*

Response 5: We have now rectified this in our revision (**Line No: 220**).

We hope the reviewer is satisfied with our responses and now finds the manuscript suitable for publication in Nature Communications.

Reviewer #3 (Remarks to the Author):

Comment: *Singh, Sood, and Ganapathy examined a bi-disperse colloidal liquid trapped on the surface of a sphere. This is an experimental realization of a two-dimensional glassy system. The curvature and the finite size of the system gives it characteristics that are different than for two-dimensional and three-dimensional systems. Two of the author's conclusions are that the mode-coupling theory describes the dynamics and that the cooperatively rearranging regions change from string-like to compact near the mode-coupling crossover. Additionally, the Mermin-Wagner fluctuations that have been determined to be very important for two-dimensional liquids are irrelevant for liquids on a sphere.*

The experiment is interesting and the analysis is well done. I have some questions about comments about the discussions and conclusions that need to be addressed before I can recommend the paper for publication.

Response: We thank the referee for finding our experiments interesting and analysis well done. Below we provide a point-by-point response to their comments.

Comment 1: *The non-monotonic evolution of the height of the first peak of of the pair correlation function is only due to one point. The authors should add another point at for Gamma greater than 80 to verify this non-monotonic evolution.*

Response 1: We have now carried out two experiments, one for $\Gamma \sim 120$ and the other for $\Gamma \sim 40$ (asked by Referee 1) and the results of these experiments, combined with the previous ones, are shown in the **Inset to Fig. 1e, Fig 2b, Fig. 3c and Fig. 4 of the Main Manuscript**. These experiments only further reinforce our findings. The high Γ experiment also helped confirm the non-monotonic evolution of the pair-correlation function.

Comment 2: *In Figure 2a, the cage relative is shorter for Gamma = 9 than the non-cage relative relaxation time, but the non-cage relative relaxation time is shorter at every other Gamma. Can the authors explain why Gamma = 9 is different?*

Response 2: We thank the referee for this comment. At small Γ, the cages around the particles are not rigid and they themselves are quite mobile. Thus, in the cage-relative measures, the cage-displacement adds a random noise to the particle over and above true Brownian motion and this results in a faster decay of the time-correlation functions. When Γ is increased, the cages become rigid and this effect diminishes and the non-cage measure decays faster than the cage measure. We have now mentioned this in the caption to **Fig. 2a of the Revised Manuscript**.

***Comment 3**: Figure 2a is hard to follow at first. By connecting the symbols with lines it takes a reader awhile to understand the figure.*

Response 3: We have now corrected this in the revision. The lines for the non-cage measure have been made thicker for enhancing clarity.

***Comment 4:** As a comment, it is unsurprising that the mode-coupling-theory provides a good fit to the first three decades of slowing down observed in these experiments since this is observed in many liquids. One generally needs several more decades of slowing down to observe deviations from the mode coupling-theory. Therefore, I don't find this observation to warrant publication in Nature Communications.*

Response 4: MCT has often been the first line of attack to describe dynamical slowing in Euclidean space and we have now shown that it is well-suited for describing dynamical slowing down on curved surfaces as well. That MCT works on curved surfaces has been demonstrated only recently in numerical studies by Viot, Tarjus and coworkers (**Ref [9] of Main Manuscript**). We would also like to emphasize that these are the first experiments on curved surfaces and our findings about MCT scaling being applicable to our system was appreciated by both Referees #1 and #2. Also, for a liquid of soft-particles with dipolar interactions, it was not a priori clear what would be the relevant parameter for carrying out the MCT scaling analysis. Our experiments have shown that the control parameter Γ is appropriate for carrying out this scaling. Also, by identifying the MCT singularity, we have shown that the shapes of CRRs become more compact in its vicinity - a key prediction unique to RFOT. This would have not been possible without carrying out the MCT scaling analysis first.

***Comment 5:** This is not the first time it has been reported that the shapes of cooperatively re-arranging regions are changing from string-like to compact near the mode-coupling crossover. This change in shape was shown in the work of Flenner, Staley, and Szamel (PRL 112, 097801, 2014). Other authors have inferred a change of shape also. These previous works should be discussed in the manuscript.*

Response 5: We apologize for this oversight. We have now included the reference suggested by the referee (**Ref [13] of Main Manuscript**) and this reference is cited when we discuss the changing morphology of CRRs in the introduction paragraph (**Line No: 26 of Main Manuscript**) and also when we discuss our results (**Line No: 190-193 of Main Manuscript**).

***Comment:** Due to the issues listed above, I do not think the authors have demonstrated that the results are novel enough for publication in Nature Communications.*

Response: Up until now, the dynamics of liquids on non-Euclidean surfaces has often been studied in the context of the Geometric Frustration-based theory of the glass transition. As we mentioned earlier, even MCT was extended only recently to such surfaces (Response 4). By experimentally observing for the first time that the CRRs shape changes from string-like to compact near the MCT singularity, we have directly verified a prediction unique to RFOT. We believe this observation will generate immense interest in attempting to reconciling our observations within other theoretical paradigms as well. This was also the specific comment made by Reviewer #2. Besides these findings, we have also experimentally explored for the first time the significance of MW fluctuations in liquids on closed surfaces. Last but not the least, these are also the first experiments probing the dynamics of bidisperse liquids on a sphere. Given the similarity in their structure and dynamics with their Euclidean counterparts, it should now be possible to explore finite-size effects in experiments without worrying about boundary conditions as there are no boundaries on a closed surface. Besides surfaces of positive

curvature, our experiments also allow the possibility of probing liquid dynamics on surfaces with negative curvature. These experiments are currently underway in our laboratory.

We hope the reviewer is satisfied with our responses and now finds the manuscript suitable for publication in Nature Communications.

REVIEWERS' COMMENTS:

Reviewer #1 (Remarks to the Author):

In the new version of the manuscript the authors have addressed in a reasonable manner the points I raised in my previous report. Therefore I do recommend the manuscript to be accepted. Below some small points that should be fixed.

l. 64: The "n" in the equation is the particle density? Correct?

l. 105: "Lenard" => "Lennard"

l. 106: "possibly due to the presence of a hexatically ordered phase at low temperatures." This might be, but the LJ potential is also much stiffer than the one considered here, thus resulting in a larger correlation length.

l. 144: "and the stretching exponent β CR systematically decreased with Γ (Fig. 2b)". I don't agree: The exponent decreases at small Gamma and then stays constant. Only once one reaches Gamma_MCT the exponent starts to drop again. So this plateau is exactly what is predicted by MCT.

l. 149: The exponent is "-gamma" and not "-1/gamma"

l. 150: "which ranges from 1.5-2 depending". This exponent can be larger than 2.0, so better replace this bound by 3.0.

Once these issues are fixed I recommend the paper to be accepted. It is very nice and interesting work.
Walter Kob

Reviewer #2 (Remarks to the Author):

The revised version properly addresses all my minor concerns and the ones of the other two reviewers. I believe this version of the manuscript is suited for publication in Nature Communications.

Reviewer #3 (Remarks to the Author):

The authors have done a very thorough and convincing job at addressing all the referees concerns. While I am still not convinced that the mode-coupling analysis is very significant, I find that I may be in the minority and concede that point. I find the revised manuscript to be suitable for publication.

Response to Reviewers' comments:

Reviewer #1 (Remarks to the Author):

Comment: *In the new version of the manuscript the authors have addressed in a reasonable manner the points I raised in my previous report. Therefore I do recommend the manuscript to be accepted. Below some small points that should be fixed.*

Response: We thank the reviewer for their suggestions, comments. These have helped significantly improved our manuscript. Below we provide a point-by-point response to the reviewer comments.

Comment: *l. 64: The "n" in the equation is the particle density? Correct?*

Response: The referee is indeed very correct. The "*n*" in the equation is particle density/areal density and is define in **Page 4, paragraph 3 of Main Manuscript**.

Comment: *l. 105: "Lenard" => "Lennard"*

Response: We apologize for this typographical mistake. We have corrected this in our revision (**Page 6, paragraph 2 of Main Manuscript**).

Comment: *l. 106: "possibly due to the presence of a hexatically ordered phase at low temperatures." This might be, but the LJ potential is also much stiffer than the one considered here, thus resulting in a larger correlation length.*

Response: This may be but we would like to point out that in a recent experiment and simulation study, Guerra and co-workers (**Ref. [4] of the Main Manuscript**) found that for monodisperse particles with dipole-dipole interaction the correlation length at high Γ (low temperature) on the surface of an S^2 sphere is indeed large. Simulations of monodisperse particles with LJ interactions on S^2 have also found a larger correlation length of almost 5σ at low temperatures (**Ref. [22] of the Main Manuscript**). Although LJ potential is much stiffer than the dipole-dipole interaction, both interactions result in a larger correlation length for monodisperse particles on the surface of an S^2 sphere. Both systems possess hexatic order at low temperature/high Γ. We therefore feel that this clear evidence for static order is the reason for the large correlation length.

Comment: *l. 144: "and the stretching exponent β CR systematically decreased with Γ (Fig. 2b)". I don't agree: The exponent decreases at small Gamma and then stays constant. Only once one reaches Gamma_MCT the exponent starts to drop again. So this plateau is exactly what is predicted by MCT.*

Response: We are grateful to the reviewer for bringing this to our notice. Following the referee's suggestion, we have now reworded this line as:
"the stretching exponent β_{CR} decreases at small Γ and develops a plateau at intermediate Γ before decreasing again near Γ_{MCT}. (**Page 7, paragraph 1 of Main Manuscript**).

Comment: *l. 149: The exponent is "-gamma" and not "-1/gamma".*

Response: We apologize for this typographical mistake. We have corrected this in our revision (**Page 7, paragraph 2 of Main Manuscript**).

Comment: *l. 150: "which ranges from 1.5-2 depending". This exponent can be larger than 2.0, so better replace this bound by 3.0.*

Response: We agree with the reviewer, and we now use the exponent(γ) ranges from 1.5-3 (**Page 7, paragraph 2 of Main Manuscript**).

Comment: *Once these issues are fixed I recommend the paper to be accepted. It is very nice and interesting work.*

Response: We hope the reviewer is satisfied with our response. We also thank the reviewer for these encouraging remarks and recommending the manuscript to be accepted in Nature Communications.

Reviewer #2 (Remarks to the Author):

Comment: *The revised version properly addresses all my minor concerns and the ones of the other two reviewers. I believe this version of the manuscript is suited for publication in Nature Communications.*

Response: We thank the reviewer for the positive assessment of our work and for recommending the manuscript for publication. We also thank the reviewer for previous suggestions and comments; they improved our manuscript.

Reviewer #3 (Remarks to the Author):

Comment: *The authors have done a very thorough and convincing job at addressing all the referees concerns. While I am still not convinced that the mode-coupling analysis is very significant, I find that I may be in the minority and concede that point. I find the revised manuscript to be suitable for publication.*

Response: We thank the reviewer for finding our responses thorough and convincing. We also thank the referee for finding the revised manuscript suitable for publication.
